# An expanded model of HIV cell entry phenotype based on multi-parameter single-cell data

**DOI:** 10.1186/1742-4690-9-60

**Published:** 2012-07-25

**Authors:** Katarzyna Bozek, Manon Eckhardt, Saleta Sierra, Maria Anders, Rolf Kaiser, Hans-Georg Kräusslich, Barbara Müller, Thomas Lengauer

**Affiliations:** 1 Department of Computational Biology and Applied Algorithmics, Max Planck for Computer Sciences, Campus E1 4 66123, Saarbrücken, Germany; 2 Department of Infectious Diseases Virology, University of Heidelberg, Im Neuenheimer Feld 324, 69120, Heidelberg, Germany; 3 Institute of Virology, University of Cologne, Fürst-Pückler-Strasse 56 50935, Cologne, Germany; 4 Current address: CAS-MPG Partner Institute for Computational Biology, Shanghai, P.R. China; 5 Current address:Department of Cellular and Molecular Pharmacology, University of California, San Francisco, CA 94158, USA

## Abstract

**Background:**

Entry of human immunodeficiency virus type 1 (HIV-1) into the host cell involves interactions between the viral envelope glycoproteins (Env) and the cellular receptor CD4 as well as a coreceptor molecule (most importantly CCR5 or CXCR4). Viral preference for a specific coreceptor (tropism) is in particular determined by the third variable loop (V3) of the Env glycoprotein gp120. The approval and use of a coreceptor antagonist for antiretroviral therapy make detailed understanding of tropism and its accurate prediction from patient derived virus isolates essential. The aim of the present study is the development of an extended description of the HIV entry phenotype reflecting its co-dependence on several key determinants as the basis for a more accurate prediction of HIV-1 entry phenotype from genotypic data.

**Results:**

Here, we established a new protocol of quantitation and computational analysis of the dependence of HIV entry efficiency on receptor and coreceptor cell surface levels as well as viral V3 loop sequence and the presence of two prototypic coreceptor antagonists in varying concentrations. Based on data collected at the single-cell level, we constructed regression models of the HIV-1 entry phenotype integrating the measured determinants. We developed a multivariate phenotype descriptor, termed phenotype vector, which facilitates a more detailed characterization of HIV entry phenotypes than currently used binary tropism classifications. For some of the tested virus variants, the multivariant phenotype vector revealed substantial divergences from existing tropism predictions. We also developed methods for computational prediction of the entry phenotypes based on the V3 sequence and performed an extrapolating calculation of the effectiveness of this computational procedure.

**Conclusions:**

Our study of the HIV cell entry phenotype and the novel multivariate representation developed here contributes to a more detailed understanding of this phenotype and offers potential for future application in the effective administration of entry inhibitors in antiretroviral therapies.

## Background

Human immunodeficiency virus (HIV) entry into host cells is initiated by binding of the viral envelope (Env) glycoprotein gp120 to the primary cellular receptor CD4 [1,2]. CD4 binding induces conformational changes in the gp120 glycoprotein [3], resulting in formation of a binding site for specific chemokine receptors, most importantly CCR5 and CXCR4 for HIV type 1 (HIV-1), which serve as coreceptors for HIV entry [4-6]. The interaction of gp120 with the coreceptor induces a series of further conformational rearrangements in the viral Env glycoproteins that ultimately result in fusion of the virus envelope with the host cell membrane [1].

It has been shown that viruses using CCR5 (R5-tropic viruses) are almost exclusively present during the early asymptomatic stage of the infection whereas CXCR4-using viruses (X4-tropic viruses) emerge in later phases of the infection in about 50% of cases and are associated with a CD4^+^ T-cell decline and progression towards AIDS [7,8]. The finding that individuals lacking CCR5 expression due to a homozygous deletion in the *ccr5* gene (CCR5/**Δ**32) are resistant to HIV-1 infection without suffering from adverse effects [9] stimulated the search for HIV inhibitory CCR5 antagonists, which culminated in the approval of the compound Maraviroc (MVC) [10] for clinical use. The correlation of viral tropism with disease progression and its significance for treatment strategies specifically targeting R5 viruses underscore the clinical relevance of accurate monitoring of coreceptor usage.

The principal viral determinant of HIV coreceptor specificity is the third variable (V3) loop of gp120 [11-13]. This is supported by several studies on the power of genotypic prediction based on the sequence of the V3 loop (see, e.g. [14-16]). Those methods have been developed as an alternative to time-consuming and expensive phenotypic assays for surveying HIV coreceptor usage of viral populations from patient’s samples. They aim at computationally predicting viral tropism based on the V3 loop sequence [11,12,17-20] and on its structure [21,22]. The straightforward accessibility of computational prediction methods and the comparatively low cost of genotyping represent major advantages of sequence-based computational approaches for predicting coreceptor usage. Due to these advantages genotypic tropism testing has entered clinical practice in Europe and has been acknowledged by the European expert guidelines on tropism testing [23]. Currently used approaches classify virus isolates into either R5- or X4-tropic based on their V3 loop sequence. The limited accuracy of current prediction methods [20] advocates the development of expanded mathematical models of virus phenotype integrating environmental and host molecular factors that are known to play a role in HIV entry in addition to the viral envelope sequence. Such models will not only contribute to our understanding of the HIV entry process, but also provide a basis for more effective therapeutic use of HIV entry inhibitors.

Numerous factors determine the efficiency of the HIV membrane fusion process. Major determinants are the amino acid sequence of the viral Env protein and the availability, and concentration of CD4, and the two major coreceptors on the cell surface. Furthermore, the presence and concentration of compounds blocking HIV coreceptors can influence virus cell entry [24]. AMD-3100 (AMD), a drug blocking CXCR4, was the first coreceptor antagonist described for HIV-1 [25], but was never approved for clinical use in HIV infected patients due to severe adverse effects [26]. In contrast, several CCR5 antagonists have entered clinical trials [27], with Maraviroc approved for patient treatment [28]. Since this drug is only effective in patients harbouring R5-tropic virus variants, viral tropism testing at the start of treatment is mandatory for prognosis of MVC therapy outcome. MVC resistance has been shown to evolve under therapy, not only through a switch of the viral population to X4-tropic variants, but also by mutations in the V3 loop enhancing the affinity of gp120 to MVC-bound coreceptor molecules, which facilitate efficient entry mediated by CCR5 in the presence of the drug [29,30]. Nevertheless, with an increasing number of patients under MVC treatment a better understanding of the evolution of the virus under coreceptor antagonist drug pressure as well as the development of algorithms accurately predicting the efficacy of entry inhibitors are highly important.

HIV entry efficiency and its sensitivity towards coreceptor antagonists are complex phenotypes simultaneously depending on multiple determinants. In this study we aimed at a more comprehensive description of this multi-variant phenotype than what is reflected in current phenotype analyses, to provide a basis for an improved prediction of HIV entry phenotypes. We present a model reflecting the multi-dimensional HIV entry phenotype based on a comprehensive experimental analysis of HIV cell entry efficiency dependent on its main determinants V3 loop sequence, cell surface CD4, CCR5 and CXCR4 expression levels, as well as on the presence of two prototypic coreceptor antagonists (MVC and AMD). Our analysis is based on an experimental assay in which natural molecular variation of the receptor and coreceptor expression on the surface of a T-cell line and its effect on viral cell entry are quantified at the single-cell level. In this system we tested a panel of isogenic viruses differing only in the sequence of the V3 loop to limit the number of biological variables and developed regression models based on the single-cell data describing HIV cell entry dependence on the measured parameters. The models provide a comprehensive representation of the viral phenotype, including effects of multiple important host and drug determinants on HIV entry and, in this way, raising tropism classification based on virus genotype to a new level of detail. Our approach offers an improved capacity of identifying X4 viruses over existing sequence-based methods for tropism prediction. It additionally allows for recognizing a spectrum of virus phenotypes extending beyond binary R5/X4 tropism classification. Given a sufficient number of variants characterized in our assay, the multivariate phenotype descriptor incorporating virus susceptibility to MVC can be inferred from V3 sequence, which is of high interest for practical use of our approach in patient treatment with coreceptor antagonists.

## Results

### Experimental and computational setup for acquisition and analysis of single-cell data

**Figure 1:**
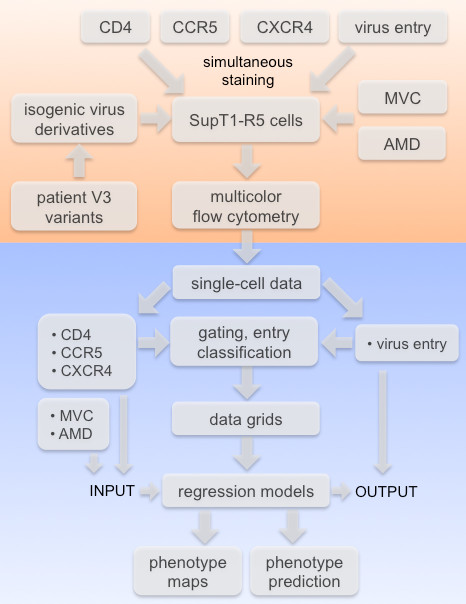
**Data acquisition and analysis.** The flow chart depicts the experimental and computational pipeline employed in this study. Steps of the experimental procedure are shown in the orange part of the plot; computational steps are shown in the blue part of the plot.

In this study we established a flow cytometry based assay for simultaneous measurement of the efficiency of HIV-1 cell entry and cell surface receptor and coreceptor expression at the single-cell level (Figure 1). We designed the experimental setup in a way intended to limit the variation of parameters which were not considered in the data collection and the subsequent modelling steps. Since primary T-cells derived from peripheral blood mononuclear cells (PBMCs) would introduce unpredictable donor specific variations beyond the level of CD4 and coreceptors on the cell surface, we employed a characterized T-cell line (SupT1/CCR5) expressing both coreceptors, CXCR4 and CCR5, as host cells. Receptor and coreceptor levels on this cell line were compared to those on primary T-cells derived from different blood donors (Additional file 1: Figure S1). Quantitative flow cytometry analysis revealed that SupT1/CCR5 cells displayed approximately two- to three-fold higher amounts of CXCR4 and CD4, respectively, on the cell surface compared to primary T-cells. This was balanced by a larger cell size of the SupT1/CCR5 cells (approximately two-fold higher volume as estimated from FSC/SSC parameters [31]), rendering the differences in CD4 and CXCR4 surface concentrations negligible. Levels of CCR5 on SupT1/CCR5 cells were approximately 6-fold higher than on PBMCs due to exogenous expression of this chemokine receptor in the cell line. Even though individual cells from this cell line are more homogeneous than primary cells isolated from different donor samples, cell-to-cell variations in CD4, CCR5 and CXCR4 levels within the population of SupT1/CCR5 covered a sufficiently wide range, enabling us to measure the effect of their expression variation on the efficiency of virus entry. Within the set of experiments used in this study, the expression levels of CD4, CCR5 and CXCR4 on the SupT1/CCR5 cell line ranged between 3,300-45,000, 700-12,000 and 3,000-44,400 molecules per cell, respectively (Additional file 1: Figure S2 and Additional file 1: Table S1). In order to correlate HIV entry efficiency with the density of CD4, CCR5 and CXCR4 on the cell surface, virus entry into individual cells was assessed in parallel with receptor- and coreceptor immunofluorescence measurements. This was done using a β-lactamase virion fusion assay based on fluorescence cytometry (BlaM assay; [32]). In this setup, β-lactamase packaged in the reporter viruses and released into the cytoplasm upon virus entry cleaves the fluorogenic dye CCF-2 that has been loaded into the host cells, leading to a shift from green to blue fluorescence emission.

The variability of tested virus variants was restricted to the amino acid sequence of the V3 loop. This loop has been described as the main predictor for viral tropism by numerous studies [11-13]. While sequences outside of the V3 region have been found also to contribute to HIV tropism [16], a high concordance between predictions based on bulk V3 sequencing of clinical samples and phenotypic tropism testing by Enhanced Sensitivity Trofile Assay (ESTA; a validated phenotypic assay) have been described [33-35] suggesting genotypic analysis of the V3 loop sequence alone as an accepted analysis method for clinical purposes [23,36]. Therefore, our analysis was performed on a panel of isogenic reporter viruses differing only in the amino acid sequence of the V3 loop.

We selected a set of 16 diverse patient-derived, and five lab-adapted V3 loop variants [37-40] based on their predicted tropism as determined by three tropism prediction tools [11,12,17,20] and sequence similarity to other V3 loops (compare Methods section; Additional file 1: Table S2). We used the X4-tropic lab-adapted strain NL4-3 [41] and its R5-tropic derivative NL4-3 R5 (kindly provided by S. Pöhlmann), differing by seven point mutations within the V3 loop as reference X4 and R5 viruses, respectively. Among the selected patient-derived sequences two were predicted as R5-tropic, 11 as X4-tropic and three showed ambiguous results in the sequence analysis (*ambiguous variants*). Among the lab-adapted strains, four were predicted as R5-tropic and one as X4-tropic (Additional file 1: Table S2). Analysis of the V3 sequences revealed that all sequences assigned as R5, as well as one ambiguous variant (924), were closely related to other R5 sequences as indicated by short branch lengths of the respective clades in the phylogenetic tree (Additional file 1: Figure S3). The reference X4 sequence, one lab-adapted X4 sequence (HxB2), and one of the patient-derived sequences predicted as X4 (286), belonged to the clades comprising predominantly X4 sequences. Among the remaining sequences, four (252, 381, 315 and 376) formed a closely related, seemingly dual-tropic group. Other sequences were located in parts of the tree occupied by both R5 and X4 sequences showing intermediate branch lengths. Detailed characterization of the sequences is provided in the Supplementary Information.

To ensure appropriate expression levels of the modified Env proteins, we introduced a gene segment encoding for the respective V3 loop into the NL4-3 proviral backbone. Reporter viruses carrying a Vpr-BlaM fusion protein were prepared from the tissue culture supernatant of transfected 293T cells and used to infect target cells at conditions yielding 20 to 30% entry positive cells. Infected cells were stained for BlaM activity as well as immunostained for cell surface expression of CD4, CXCR4 and CCR5. All parameters were subsequently assessed in parallel on the single-cell level by multicolour flow cytometry.

An automated data analysis procedure was developed in order to process large amounts of data sets and eliminate investigator bias through manual gating of flow cytometry data. This included automated gating of the major live cell population based on forward scatter (FSC) and side scatter (SSC) flow cytometry values (Additional file 1: Figure S4), followed by binary classification into *entry positive* and *entry negative* using a computationally established decision boundary based on control measurements performed in parallel on uninfected cells (Methods; Additional file 1: Figure S5). This procedure was validated by manual gating of a subset of measurements (R^2^ = 0.997; Additional file 1: Figure S6).

### Analysis of multivariate data sets and comparison to binary sequence classification results

**Figure 2:**
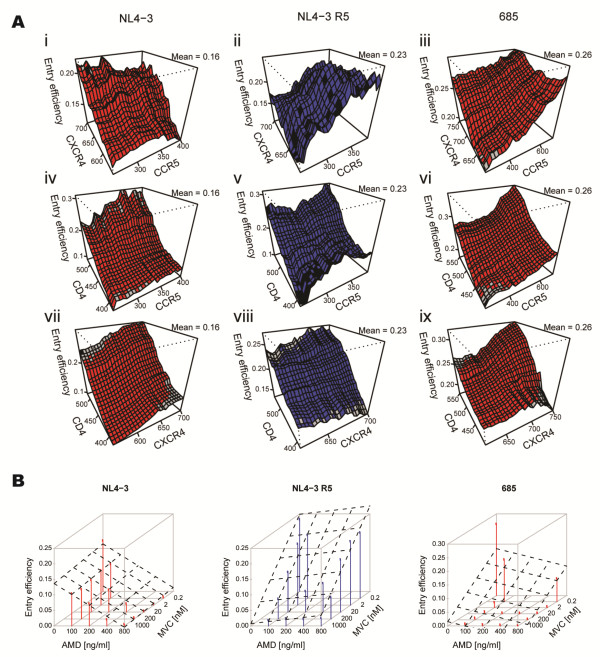
**Exemplary illustrations of the dependence of viral entry efficiency on various tested parameters.** The figure shows a graphical representation of exemplary data collected for the two reference clones (NL4-3 and NL4-3 R5) and one dual-tropic clone (685). Dependence of the entry efficiency on the surface expression levels of all receptor/coreceptor combinations (**A**) and on concentrations of two entry inhibitors (**B**) from representative experiments are shown. Concentrations of AMD and MVC are shown in ng/ml and nM, respectively. Planes traced with dashed lines were fitted using linear regression and indicate the direction of the decrease of the entry efficiency with the increase of each drug concentration.

The measurements on the single-cell level and their automated classification resulted in data sets comprising information on relative CD4, CXCR4 and CCR5 expression levels for each individual cell, in conjunction with the binary information whether reporter virus had entered the respective cell. Analogous data sets were collected in the presence of varying concentrations of AMD and MVC. This set of measurements was performed for each virus variant. Figure 2 illustrates the multi-dimensionality of the phenotype measured in this study. Panels A depicts the entry efficiency of the reference X4 and R5 variants and one exemplary variant with a patient-derived V3 loop (685), respectively, dependent on different expression levels of all receptor and coreceptor combinations in the absence of drugs. Entry of the reference X4 virus NL4-3 depended predominantly on the expression level of CXCR4 as reflected by the gradient of the diagram depicting entry efficiency in dependence of both coreceptors (Figure 2A i). In contrast, the reference R5 virus NL4-3 R5 showed dependence predominantly on the expression level of CCR5 (Figure 2A ii), and virus 685 on the expression levels of both CCR5 and CXCR4 coreceptors (Figure 2A iii). For a better understanding of these complex dependencies, the entry efficiency in dependence of either one of the coreceptors at various CD4 expression levels is depicted on the remaining plots of this panel (Figure 2A iv-ix). Analysis of entry efficiency in dependence of CXCR4 and CD4 showed a clear R5 tropic phenotype of the NL4-3 R5 variant with no apparent dependence on CXCR4 (Figure 2A viii). In contrast, virus 685 did display dependence on both CCR5 and CXCR4 (Figure 2A vi and ix). These visualizations reveal the degree of molecular variability within a cell population considered to be homogeneous, and its effect on virus cell entry which is masked in bulk analyses. Figure 2B illustrates the dependence of viral entry efficiency on both coreceptor antagonists MVC and AMD present at different concentrations. Sensitivity of the reference viruses NL4-3 and NL4-3 R5 towards the CCR5 and CXCR4 antagonists, respectively, was in accordance with their coreceptor dependence. Virus 685 displayed a more complex phenotype, showing reduced sensitivity towards AMD and complete inhibition only in presence of both coreceptor antagonists (Figure 2B, right panel).

**Figure 3:**
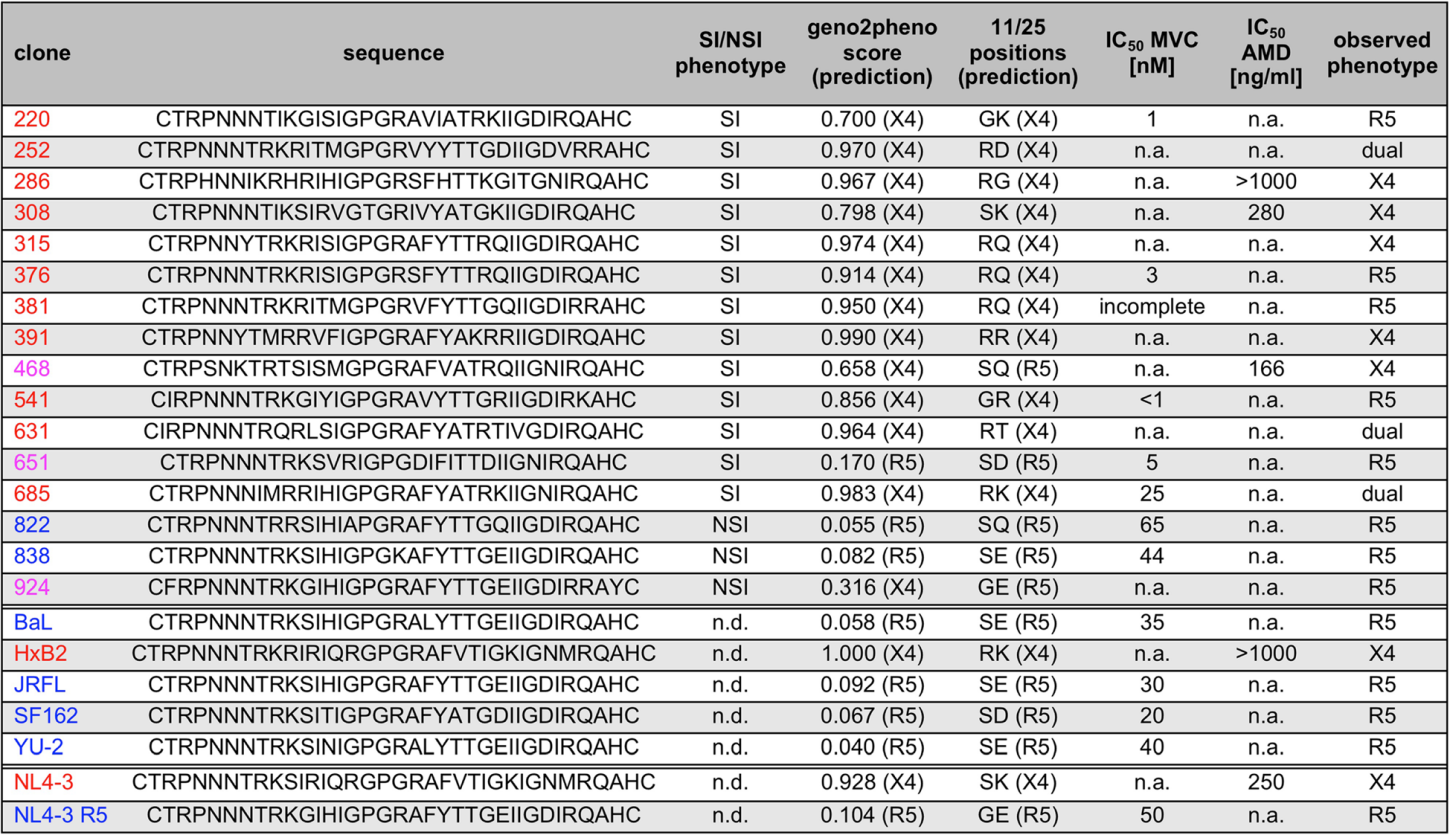
**Characterization of V3 sequences of clones tested in this study.** Column “phenotype” indicates syncytium (SI) or non-syncytium (NSI) inducing phenotype as determined on PBMC cells; n.d., not determined. Columns “geno2pheno score” and “11/25 positions” indicate the prediction score of geno2pheno[coreceptor] method [20] and 11 and 25 residues [11,12], and the tropism predicted based on these two methods, respectively. A false positive rate of 5% was selected as the decision cut-off for geno2pheno prediction. The colour indicates the predicted phenotype: blue– clearly R5, red – clearly X4, magenta – ambiguous. To obtain IC_50_ values, entry efficiency was determined by bulk analysis in presence of serial dilutions of the respective coreceptor antagonist as described in Methods. Dose response curves were fitted using GraphPad Prism software. Mean values of three independent experiments are depicted. n.d., not determined in our analysis; n.a., not applicable (no dose response curve could be fitted to the data due to lacking inhibition by the respective drug); incomplete, steep dose response curve with reduced maximum inhibition. “Observed phenotype” indicates the clone phenotype observed in our assay based on the sensitivity to AMD and MVC and entry dependency on the two coreceptors.

Initial visual inspection of the experimental data of all tested virus variants allowed us to distinguish X4, R5 and dual-tropic viruses in our dataset (Figure 3). Among 11 V3 loop sequences classified as X4 based on the sequence analysis, only four showed a clear X4-tropic phenotype in our phenotypic analysis, as demonstrated by sensitivity towards AMD and dependence on CXCR4 expression levels. Three of the remaining seven viruses displayed an R5-tropic phenotype characterized by sensitivity to MVC and dependence on CCR5 expression, and the four remaining viruses showed a dual tropic phenotype characterized by dependence on the levels of both coreceptors and response to combinations of coreceptor antagonists. In contrast, none of the variants classified as R5-tropic showed an X4-tropic phenotype in our assay. From three viruses initially classified as ambiguous, two showed R5- and one X4-tropic characteristics. Notably, the incorrectly predicted variants showed stronger response to MVC, even at low drug concentrations, than viruses initially classified as R5-tropic. In summary, from 19 clearly classified V3 sequences, only 11 were confirmed in their predicted tropism, two showed an opposite tropism and four displayed dual tropism. Three variants classified as ambiguous displayed clear R5- or X4-tropic phenotypes in our assay (Figure 3). Taken together, these results reflect a higher accuracy of detection of X4 phenotypes by our multi-parameter phenotypic assay as compared to the NSI/SI phenotypic and genotypic classification methods used traditionally to characterize the V3 sequences, and allowed characterization of complex phenotypes for which these tools proved insufficient.

### Phenotype maps

**Figure 4:**
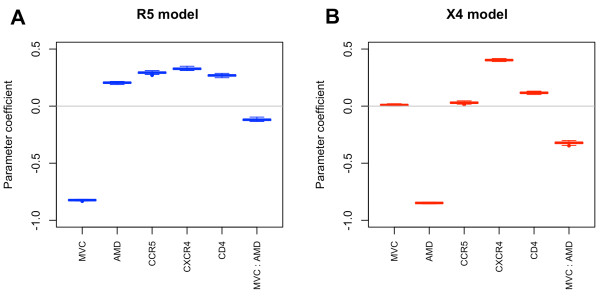
**Variable coefficients of reference models.** Boxplots show parameter coefficient variation for R5 (**A**) and X4 (**B**) reference models in a bootstrapping test of model training on 75% data points sampled 100 times out of the entire set of data.

Data sets collected on the single-cell level were used to construct regression models reflecting dependence of the virus cell entry efficiency on the measured parameters. In these models, CD4, CCR5 and CXCR4 surface levels as well as MVC and AMD concentrations were used as input variables, while virus entry into a cell, as determined by the BlaM assay, was used as the output variable. Prior to model training, the single-cell data obtained with each of the variants were combined into a multidimensional grid defined on values representing CD4, CCR5 and CXCR4 expression levels aggregated into value segments and on all tested drug concentrations. Virus cell entry information was averaged over the cells within each bin of the grid. Model selection was performed on a variety of model classes (Additional file 1: Table S3) defined on the five input variables and based on the data grids of the two reference viruses (NL4-3, NL4-3 R5). The best models were chosen according to two criteria: (i) the *model fit* to the data and (ii) the *model separation* between the X4 and R5 reference models (NL4-3 and N4-3 R5, respectively). Since in this study models of cell entry efficiency were used as a description of a complex virus phenotype rather than for predicting the entry efficiency for a specific virus, we minimized the training error rather than the test error that quantifies model predictive power. Among 192 analyzed model classes two models were selected as the best representation of the experimental data obtained for both reference viruses (Additional file 1: Figure S8). These models differ in the scale of the MVC and AMD input variables, with a logarithmic scale offering the better model fit (R^2^ ~ 0.62 vs. 0.49) and a linear scale offering the better model separation (R5-X4 model distance of about 1.94 vs. 1.74) (Additional file 1: Table S5) and were termed *logarithmic model* and *linear model* respectively. Since the linear model produced a better separation of the reference models, we focused on results based on the linear model in the following parts of this study. Details of the model selection and the comparison of the R5 and X4 models are provided in the Supplementary Information (Additional file 1: Table S3, Additional file 1: Table S4, Additional file 1: Table S5 and Additional file 1: Figure S8). Next, the selected model was trained on the data grids of all tested viruses separately. The vector of parameter coefficients of each virus model was extracted and used as a multivariate descriptor of the phenotype, termed here *phenotype vector*. The phenotype vectors of the reference viruses NL4-3 and NL4-3 R5 represent reference X4 and R5 phenotypes, respectively. The magnitude and sign of the respective parameter coefficient describe the phenotype of a virus variant in terms of its receptor and coreceptor usage and its susceptibility to the two coreceptor antagonists (Figure 4). Input variable coefficients describing the reference models varied mostly in the respective drug and coreceptor coefficients and showed comparable CD4 dependence of both virus tropisms as reflected in comparable input variable coefficients for this parameter.

**Figure 5:**
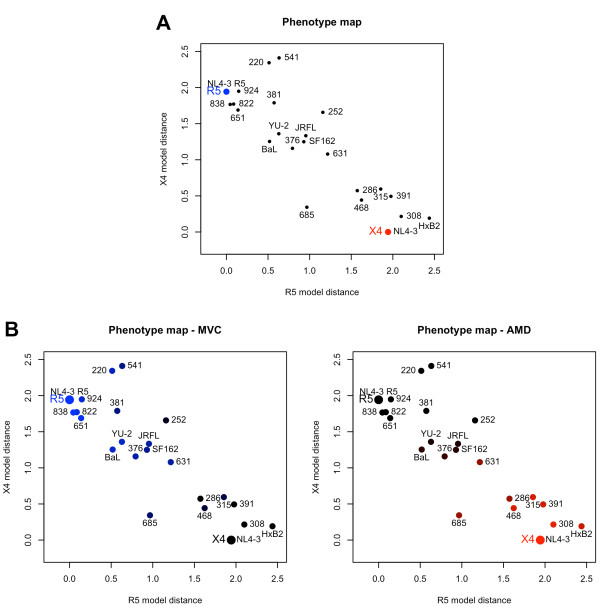
**Phenotype maps.** (**A**) Clone phenotypes are represented by dots; reference phenotypes are marked with larger red (X4) and blue (R5) dots. (**B**) Phenotype maps coloured according to the MVC (left-hand panel) and AMD (right-hand panel) coefficients. In the left-hand panel the colour ranging from blue to black indicates the size of the MVC coefficient with a small coefficient indicating susceptibility to MVC coloured in blue. In the right-hand panel, the colour ranging from red to black indicates the size of the AMD coefficient with a small coefficient indicating susceptibility to AMD coloured in red. Phenotype maps shown in this figure are based on the linear phenotype model, see Supplementary Information (Additional file 1: Figure S9) for the phenotype map based on the logarithmic model.

The Euclidean distance between the phenotype vectors of the tested variants and that of each of the two reference phenotypes was used as a measure of similarity to both reference phenotypes. Thus, each variant was represented by two values expressing its similarity to the R5- and X4-tropic phenotype, respectively. This representation allows for visualizing the phenotype in convenient 2D plots, termed here *phenotype maps* (Figure 5). The proximity of variants in the phenotype maps to the reference viruses reflects similarity of entry efficiency phenotypes (Figure 5). Viruses located in proximity of the R5 reference model showed dependence on CCR5 expression and sensitivity to MVC, viruses located close to the X4 reference model showed dependence on CXCR4 expression and sensitivity to AMD. Viruses 685, 252 and 631 showed a dual-tropic phenotype in our assay and were located in the central area of the map, distant from the two reference models. The different degrees of similarity of the variant phenotypes to the reference phenotypes are reflected by their dispersed position on the map illustrating how our approach allows to differentiate a spectrum of virus phenotypes which is only partially captured by binary tropism classification.

Information on the sensitivity of virus derivatives towards coreceptor antagonists is included in the phenotype vector and can be visualized on the phenotype maps. Colours on the phenotype maps in Figure 5B illustrate the strength of the inhibition by MVC and AMD. Shades of blue represent sensitivity to MVC, shades of red represent sensitivity to AMD. Black colour on both plots indicates lack of sensitivity to either of the receptor antagonists tested. Two groups of variants can be distinguished on the map, variants responsive to MVC and variants responsive to AMD, respectively (coloured in different shades of blue and red, respectively). The colouring of variants 685, 631 and 252 indicates either lower sensitivity (685 and 631) or no response (252) to both of the drugs. These three variants are also positioned between the two groups of R5 and X4 viruses on the map, variants 252 and 631 closer to R5-, variant 685 closer to X4-tropic viruses. This illustrates how the position in the map reflects the sensitivity of the variant to coreceptor antagonists and where a potential boundary between phenotypes can be located. The phenotype maps can therefore be used for visual display of the sensitivity to MVC treatment. In this way, phenotype vectors represent the large and multi-parameter single-cell data set in a quantitative and concise manner.

### Prediction of the phenotype vector

The phenotype vector provides an insightful characterization of the virus phenotype including coreceptor usage, sensitivity to drugs and its location in the spectrum of phenotypes between R5 and X4 reference phenotypes. Given the cost and duration of experimental testing of individual variants, computational prediction of virus phenotype from sequence is crucial for the practical use of the presented approach in a clinical setting. Therefore, we developed computational methods for predicting the multivariate phenotype vector based on the V3 loop sequence. Since the small number of variants presents a major limitation for the development of an accurate prediction method, we extrapolated the effect of the size of the experimentally characterized variants in the training set on prediction accuracy.

Prediction models were trained on the binary sequence encoding of the variants´ V3 sequences with the respective phenotype vectors as the output variables. To construct prediction models, we used shrinkage methods that allow for reducing the high number of dimensions of the sequence data in the presence of a low number of observations: the 23 variants tested in our study exhibited 88 positions that differed among the variants in the binary sequence encoding of their V3 loop. Among the statistical modelling approaches that we tested ([42,43]; Methods), Lasso regression [43] yielded the highest accuracy and was used in the following steps of this analysis (Additional file 1: Figure S10).

**Figure 6:**
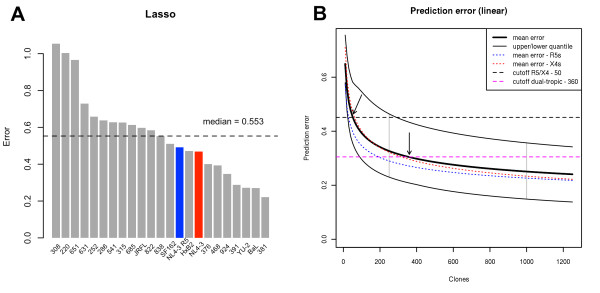
**Extrapolation of data volume needed for prediction of the phenotype vector from V3 sequence.** (**A**) Error of the predicted phenotype vectors. Predictions are calculated via LOOCV, error is estimated by the Euclidean distance between the predicted and observed phenotype vectors. Blue and red bars mark R5 and X4 reference clones, respectively. (**B**) Estimation of the training set size effect on the prediction error. Error functions were fitted to simulated training set sizes (2-22) for all tested clones (thick black line), and for the subsets of X4 (dashed red line) and R5 (dashed blue line) clones, respectively. Thin black lines represent the top and bottom 0.25 quantiles of the averaged error function for all clones with two vertical gray lines indicating the distance between the quantiles. Dashed horizontal lines represent cut-offs for recognizing R5/X4 (black) and dual-tropic (magenta) viruses, respectively. The training set size of the averaged function at two cut-offs are pointed to by arrows and indicated in the legend.

The prediction error of the phenotype vectors was estimated in terms of the Euclidean distance between observed and predicted phenotype vectors in a leave-one-out cross validation (LOOCV) on the set of tested viruses (Figure 6A). On average, X4-tropic variants showed a slightly, but not significantly, higher error than R5-tropic variants (0.562 vs. 0.525 respectively, p ~ 0.5). The highest average error was observed for dual-tropic variants (0.666), reflecting the difficulty of predicting this phenotype. Three variants (308, 220, 651) showed a markedly elevated prediction error compared to the other variants in the dataset. Notably two of these (220, 651) displayed a phenotype in our assay that was inconsistent with tropism prediction by common genotypic methods – both showed an R5 phenotype in the assay despite being previously classified as X4 (220) or ambiguous (651). V3 loop sequences of these variants were located in a sparse cluster of both R5 and X4 sequences (Additional file 1: Figure S3) suggesting that the sequences of these V3 loops represent boundary cases between two tropisms which contributes to the difficulty in their tropism prediction.

We next performed a simulation test aimed at inferring the relationship between the size of the training set and the accuracy of phenotype prediction. For each of the variants we fitted a polynomial error function $f(x)=ax^{b}$, reflecting the decrease of the prediction error with the increasing size of the training set sampled from the remaining variants in the dataset (Additional file 1: Figure S11). Three of the tested variants – 220, 651 and 308 – showed an increasing error function (b>0) suggesting that an accurate prediction of their phenotype is not possible based on the current set of tested variants. Notably these three variants showed the highest prediction error in the LOOCV test (Figure 6A) and appeared to be located in regions of V3 sequence space that are sparsely populated with sequences tested in our assay (Additional file 1: Figure S3). This indicates that they represent outliers in the sequence-phenotype pairings in our dataset, which resulted in poor accuracy of their phenotype prediction. We excluded these three variants from further error estimation tests, noting that the averaged error and the inferred training set size might be underestimated due to their exclusion. We provide a quantitative characterization of these three variants with respect to their location in sequence space in the Supplementary Information (Additional file 1: Table S6; Additional file 1: Figure S12). The parameter *b* of the functions of the remaining variants was located in the range (-0.454, -0.059) with a mean of -0.244. The mean was comparable for the R5 and X4 variants (-0.256 and -0.261, respectively), suggesting that our prediction approach allows for achieving comparable sensitivity and specificity of prediction on both kinds of variants.

Next, the fitted functions were used to approximate the prediction error of models constructed on training sets of sizes exceeding the 23 virus derivatives tested in this study. Figure 6B shows averaged error functions of all variants (thick black line), of R5-tropic variants (dashed blue line), and of X4-tropic variants (dashed red line) plotted in the range of training set size of (0, 1250] virus variants. To define the extrapolation cut-off, we introduced the notion of a *borderline phenotype*. A borderline phenotype is derived from the observed phenotype by setting the coefficients characterizing the given phenotype to zero. For an R5 virus these coefficients are associated with CCR5 and MVC levels, for an X4 virus with CXCR4 and AMD levels, for a dual-tropic virus with MVC and AMD levels. Borderline phenotypes derived in this way are located in the region between R5- and X4- tropic viruses on the phenotype map (Additional file 1: Figure S13) and represent the minimal change in a virus phenotype vector that produces misclassification in terms of virus tropism and drug response. The vector distance between the observed phenotypes and respective borderline phenotypes represents an upper bound on the permitted prediction error that produces a reliable prediction of viral tropism. We defined two cut-offs: the minimal distance to their borderline phenotype for the variants with an X4 or R5 phenotype (*R5/X4 cut-off*) and the minimal distance to their borderline phenotype for dual-tropic variants (*dual-tropic cut-off*). Dual-tropic variants are located between the R5 and X4 phenotypes in the phenotype map, the difference between their observed and borderline phenotypes is therefore small, and using it as an upper bound for prediction error represents a conservative criterion. R5 and X4 variants differ more substantially from their borderline phenotypes. Thus the error margin admissible for their correct tropism prediction is larger. The R5/X4 cut-off, therefore, represents a less stringent criterion than the dual-tropic cut-off, that might result in an incorrect prediction of the dual-tropic variants. R5/X4 and dual-tropic cut-offs are represented by dashed horizontal lines coloured in black and magenta, respectively, in Figure 6B. The averaged error function intersects the R5/X4 cut-off at the training set size of 50 and the dual-tropic cut-off at the training set size of 360. The upper 0.25-quantile of the fitted functions (black thin line in Figure 6) intersects the R5/X4 cut-off at the training set size 290. These results indicate that a training set of less than 100 variants allows for accurate prediction of R5 and X4 phenotypes. To achieve a reliable prediction of dual-tropic phenotypes the experimentally validated training set would have to be expanded to 300-400 variants.

## Discussion

The difficulty of predicting the outcome of MVC therapy in HIV-1 infected patients [29,30,44], the flexibility of the virus to use other coreceptors than CCR5 or CXCR4 [45] and the high variability of V3 loop sequences indicate that the HIV-1 cell entry phenotype is more complex than what is reflected in the binary classification of CCR5/CXCR4 coreceptor usage. In this study we address the shortcomings of current sequence-based methods of HIV tropism classification and the need for more accurate recognition of X4-tropic viruses and for better virus characterization for the effective use of coreceptor antagonists.

The study is based on multivariate datasets acquired at the single-cell level. The analysis at the single-cell level affords higher level of detail than the approach based on averaged measurements of entire cell populations [46] and provides additional information used for construction of an expanded representation of the viral phenotype. Furthermore, the integration of individual cells covering a wide range of receptor and coreceptor surface levels results in robustness of the model, making it applicable to target cell populations with different average expression levels of these proteins (Additional file 1: Figure S14). Novel methods for analysis and visualization of the large and multi-parameter single-cell data as well as for constructing comprehensive models of HIV cell entry phenotypes were developed. Our experimental and analytical approach has a higher capacity of detecting X4-tropic viruses than common sequence-based prediction methods. Several variants classified as X4-tropic or of dubious tropism based on genotypic and phenotypic tropism prediction methods showed an R5 phenotype in our assay. We observe a stronger response to MVC even at low drug concentrations of these incorrectly predicted variants than of the viruses correctly classified as R5-tropic. Thus, a weaker binding affinity to the CCR5 coreceptor might be a reason for the misclassification of these variants in the available assays used for tropism prediction.

We propose a novel representation of the HIV entry phenotype in the form of a multivariate phenotype vector expressing virus dependence on several molecular and environmental determinants. The phenotype vector and its position in the phenotype map provide a more detailed characterization of viral tropism than the binary classification. The phenotype map (Figure 5) represents a convenient visual display of virus phenotypes grouping phenotypes of similar response to coreceptor antagonists. Dual-tropic variants (252, 631 and 685) are located in the central part of the phenotype map between R5- and X4-tropic variants. Populating the map with a larger number of dual-tropic viruses will allow for establishing a boundary between the phenotypes and discriminating between viruses susceptible to either CCR5 or CXCR4 antagonists.

The experimental procedures for multi-parameter phenotypic testing for entry efficiency are costly and time-consuming and thus not applicable in a clinical setting. Therefore, one aim of this study was to explore the perspectives of virtualizing the phenotype vector by providing a computational procedure that estimates the phenotype vector based on the V3 loop sequence of the virus (see also [47]). We developed a method for predicting the phenotype vector that is based on the V3 loop sequence. The method shows comparable performance on R5- and on X4-tropic viruses suggesting that, despite the high variability of X4 sequences [48], a method of comparable sensitivity and specificity for both R5- and X4-tropic viruses can be obtained. The accurate prediction of both the X4 phenotype and the virus response to MVC, being a part of the phenotype vector, are of high interest for successful application of therapies based on coreceptor antagonists. An accurate prediction method relies on virological data describing a sufficiently large number of tested variants. We extrapolated the number of variants necessary to be tested for the derivation of accurate models for phenotype vector prediction. Our analysis indicates that fewer than 100 variants are sufficient for a reliable phenotype prediction of R5 and X4 viruses and we are currently generating data sets to accomplish this aim. Increasing the training set size to data sets experimentally determined for 300–400 variants would allow for accurate prediction of dual-tropic viruses.

## Conclusions

This study represents a step towards constructing a comprehensive computational phenotype of the HIV cell entry incorporating the strength of the effect of several molecular and environmental factors on the virus cell entry beyond binary classification of its coreceptor usage. Given the limitations of sequence-based methods of virus tropism classification, expanding models with other than sequence molecular information represents a potentially promising avenue for improving our understanding of the phenotype and its prediction.

## Methods

### Variant selection

Viral variants were selected for experimental testing from a set of 94 V3 sequences of therapy-naïve patients. Viruses from the blood samples from the Bonn hemophiliac HIV cohort [49] were cultivated in PBMCs for one month and their NSI/SI phenotype was determined by light microscopy as described in [50]. Viral RNA was isolated and sequenced as described in [51]. The obtained V3 loop sequences were characterized with respect to their location in V3 sequence space [48], NSI/SI phenotype and tropism predicted by three computational methods: geno2pheno[coreceptor] [20], WebPSSM [17] and the 11/25 rule [11,12].

### Plasmids and cell lines

The V3 loop coding sequence was amplified by PCR from the RT-PCR products of patient samples or by PCR from plasmids comprising *env* sequences of lab-adapted strains, respectively. Primers used for amplification introduced *Pvu*II (5’: 21 bp upstream of V3 loop sequence) and *Xba*I (3’: 6 bp downstream of V3 loop sequence) restriction sites, respectively. For some of the constructs, synthetic gene fragments encoding V3 loops with the respective flanking sites were used (GeneArt AG, Germany). Fragments were subcloned into pCAGGS.NL4-3-Xba, an Env_NL4-3_ expression construct derived from pCAGGS.NL4-3 (kindly provided by S. Pöhlmann) harbouring unique *Xba*I and *Pvu*II restriction sites flanking the V3 loop encoding sequence. The silent mutation generating an *Xba*I site was introduced by overlap PCR using primers introducing an A to T and a G to C conversion at positions 994 and 995 of the *env* coding sequence, respectively. To yield the respective pCHIV derivatives, a *Stu*I/*Xho*I fragment (comprising part of *env* including the V3 loop) was transferred into pCHIVΔStu, a derivative of pCHIV [52], carrying a unique *Stu*I site within the *env* gene. pCHIV derivatives encode for a non-infectious proviral NL4-3-based clone which expresses all viral proteins except the accessory protein Nef. A variant of pCHIV harbouring a frameshift mutation in the beginning of the *env* gene (pCHIV.Env-) [52] was used to produce isogenic viruses lacking the Env protein on their surface (*Env-*).

The coding sequence of an optimized version of a β-lactamase was amplified from pJH_SSbla_BlaFL_UDG_S1-1 [53] by PCR with primers introducing *Kpn*I and *Eco*RI restriction sites. The resulting fragment was subcloned into pMM310 [54] to yield pBlaM.opt, encoding for a fusion protein including the HIV-1 accessory protein Vpr and β-lactamase, separated by a recognition site for the HIV-1 protease.

293T [55], cells were grown in Dulbecco’s modified Eagle’s medium (DMEM GlutaMAX; Invitrogen) supplemented with 100 U/ml penicillin, 100 μg/ml streptomycin and 10% fetal calf serum (FCS) at 37°C, 5% CO_2_. SupT1/CCR5 cells [56] were kept in RPMI1640 GlutaMAX™ supplemented with 100 U/ml penicillin, 100 μg/ml streptomycin and 10% FCS. Transfections were carried out using polyethyleneimine according to standard procedures.

### β-lactamase virion fusion assay

Entry efficiency was determined by a previously described HIV fusion assay [32]. Briefly, viral reporter particles were prepared from 293T cells co-transfected with the respective pCHIV derivative and pBlaM.opt (plasmid ratio 15:1). At 44 h post transfection, tissue culture supernatants were precleared by filtration through a 0.45 μm nitrocellulose filter and virions were purified by ultracentrifugation through a 20% (w/v) sucrose cushion. Proteins from pelleted particles were separated by SDS-PAGE (acrylamide:bisacrylamide 200:1, 17.5% acrylamide) and transferred to a nitrocellulose membrane by semi-dry blotting. Membranes were probed with polyclonal antisera raised against HIV-1 CA. Bound antibodies were detected by quantitative immunoblot using a LiCor Odyssey system and particle concentration was determined by comparison to purified CA protein analyzed in parallel. Adjusted amounts of virus that yielded about 30% of infection (as determined by titration experiments for each virus batch) were used to infect 1x10^6^ SupT1/CCR5 cells seeded in V-bottom 96-wells. For experiments including coreceptor antagonists, cells were preincubated with varying concentrations of drugs for 1 h at 37°C before virus was added. Following incubation with virus at 37°C for 6 h, supernatant was removed and cells were incubated with the β-lactamase cleavable dye CCF2-AM (GeneBLAZER, Invitrogen) in staining medium according to the manufacturer’s instructions for 16 h at room temperature. Cells were fixed with 3% PFA/PBS for 1 h at room temperature and stained for receptor and coreceptor surface levels with monoclonal antibodies directly coupled to three different fluorophors (αCD4-APC-H7, clone RPT-4, αCD184-APC, clone 12G5 and αCD195-PE, clone 2D7/CCR5; BD Biosciences, Germany) for 1 h at room temperature. 100’000 cells per sample were analysed by flow cytometry on a BD FACS CantoII machine using FACSDiva Software. FCS2.0 files including the appropriate values for compensation (as determined by single-stained controls) were exported and subjected to computational analysis. To establish the entry positive gate, mock infections (*no virus*) were carried out in parallel for each experimental condition. In addition, viruses lacking the Env protein on their surface (*Env-*) were used in parallel to control for background signal independent of Env-receptor interactions.

### Automated gating

In an initial step of data preprocessing, cell populations were filtered according to the FSC and SSC parameters (*gating*) to identify the major cell population and filter out cells not belonging to the major population as potentially defunct or of a different cell type. In order to address the shortcomings of the classical manual gating procedure and to obtain reproducible results efficiently, we developed an automated gating procedure. Cell populations are commonly represented by 2D scatter plots with FSC along the x-axis and SSC along the y-axis. In the automated gating procedure cells were first filtered through a user-defined square window defined on the FSC and SSC values. Here, we used a filter of 300 < FCS < 1200 and 0 < SSC < 900 based on visual inspection. Next, a 2D grid of FSC and SSC values was defined and the numbers of cells in each bin of this grid were calculated, which can be presented in form of a heat map (Additional file 1: Figure S4). Each bin of the grid had width and height 5, a value that was chosen among several others based on its gating agreement with the manual method. Next, for each of the values $x_{i}$ on the x-axis (FSC) a grid bin position $\left(x_{i},y_{i}\right)$ was found that contained the maximum number of cells among all bins at the given $x_{i}$. For each such bin position $\left(x_{i},y_{i}\right)$ the minimum distance $d_{i}$ was determined such that no cells were found in the bins at equidistant positions $\left(x_{i},y_{i}-d_{i}\right)$ and $(x_{i},y_{i}+d_{i}).$ The positions $\left(x_{i},y_{i1})=(x_{i},y_{i}-d_{i}\right)$ and $\left(x_{i},y_{i2})=(x_{i},y_{i}+d_{i}\right)$ were termed *surrounding points*. Next, to produce a smooth gating line, the y-coordinates of the surrounding points were averaged: $y_{i,k}$ was replaced by the mean value of itself and two neighbouring surrounding points $\frac{1}{3}\sum_{j=i-1}^{j=i+1}y_{jk}$, for k∈{1,2} The same procedure was repeated along the y-axis. A gate was defined as the minimal contiguous area on the FSC-SSC grid encircled by the line connecting consecutive surrounding points. Example results of the automated gating procedure are shown in Additional file 1: Figure S4. The mock infection measurement *no virus* was used as the control for establishing the gate. All measurements in the same experiment were gated accordingly. For reading the FCS2.0 files the R package *prada* was used, a part of Bioconductor [57].

### BlaM classification

A classical approach to BlaM-based entry classification entails a manually established decision boundary established based on the *“no virus”* control. The decision boundary is determined based on the plot of the intensity of blue (x-axis) against green (y-axis) CCF2 signal of the cells in the control measurement. It delineates the region of uninfected cells as a minimal region cells such that y>x and ~0.01% of the control cells are located outside of this region. Measurements of cells incubated with virus variants are classified according to this control-based decision boundary.

In order to efficiently classify the large number of flow cytometry measurements and to obtain reproducible results we established an automated method of BlaM classification. Our approach is based on a linear function y=ax+b fitted to the blue (x) against green (y) CCF2 signal intensities of the cells of two merged controls – *no virus* and *unstained*. For each point on the fitted line $\left(x_{i},y_{i}\right)$, where $y_{i}=ax_{i}+b$ and $x_{i}=\text{1.1200}$, data point $\left(x_{p_{i}},y_{p_{i}}\right)$ was found such that $y_{p_{i}}<x_{p_{i}}$ that is the most distant from$\left(x_{i},y_{i}\right)$, located on the line perpendicular to the fitted line that intersects the fitted line at $\left(x_{i},y_{i}\right)$:$y=-\frac{1}{a}x+b_{i}$, where $b_{i}=y_{i}+\frac{1}{a}x_{i}$. Points $\left(x_{p_{i}},y_{p_{i}}\right)$ represent cells showing the highest shift in the blue signal relative to the green signal among the cells of the control measurements. Next, the distances of these points from the fitted line were smoothed using a sliding window approach by averaging the values within each window and adding of one standard deviation of the values within that window. The added standard deviation represents a margin beyond the control cells that ensures the required low proportion of false positives in the control measurement (~0.01%). A window size of 30 was selected as the size resulting in the best classification performance. The smoothed points projected back onto the plot of green and blue signals defined the cut-off decision line – cells represented by data points located in the part of the plot below the decision line were classified as *entry positive*, those located above the line were classified as *entry negative* (Additional file 1: Figure S5). The method design and the choice of parameters were guided by the comparison with manual classification with the goal of achieving the highest agreement on a large number of measurements (Additional file 1: Figure S6). Classification based on this procedure termed here *binary classification* assigns a binary value to each cell, 0 representing entry negative and 1 entry positive cells. To compensate for differences between the automated binary and manual classification, we additionally developed an alternative *margin classification* (Supplementary Information; Additional file 1: Figure S7). However, use of this classification method did not affect accuracy of regression models of the virus entry efficiency, and binary classification was chosen as the less complex approach throughout this study. An example of classification of virus measurement using both methods is depicted in Additional file 1: Figure S7. The steps of the classification procedure are described and illustrated in detail in the Supplementary Information.

### Visualization methods

The experimental results were depicted as 3D plots of virus entry efficiency in dependence on two chosen parameters. Colours of the plots represent the predicted phenotype of a variant – red for X4, blue for R5, magenta for variants of questionable tropism. Individual cells were localized in a 30x30 grid of values spanning the ranges of values of the two parameters. Virus entry efficiency was calculated as the fraction of infected cells (assigned using binary classification) within each bin of the grid. Prior to plotting, the grid was smoothed by averaging values from neighbouring bins of the grid. In order to account for the differing numbers of cells that show a given combination of parameter values, parts of the grid that contained less than a selected minimum number of cells are coloured in gray. The selected minimum number of cells is 10% of the expected number of cells assuming an even distribution of cells over the grid.

### Merging the data

To compensate for potential noise in the measurement at the single-cell level, the data was aggregated into a multidimensional grid defined on aggregated values of CD4, CCR5 and CXCR4 and on all measured drug concentration levels. CD4, CCR5 and CXCR4 expression levels were first scaled to standard normal distribution and cells in the top and bottom 2.5% tails of the distribution were removed. Next, a five-dimensional grid was defined – spanning all tested concentration levels of AMD and MVC and a predefined number of values of the CD4, CCR5 and CXCR4 expression levels, separating their range of expression into bins of equal size. We tested four bin sizes – 5, 10, 20, 50 – for the quality of the resulting models. Values of binary classification of individual cells were averaged in each grid bin.

Data grids of cell entry measurements of each tested virus constructed in this way were merged across the individual experiments resulting in a multidimensional data grid describing each virus’ cell entry efficiency comprehensively across all experiments.

### Model selection

The selection criteria for model quality comprised two aspects: accuracy of model fit to the data and separation of the X4 and R5 phenotype vector. The first criterion – accuracy of model fit to the data – was based on the R^2^ measure estimated as:

(1) $$R^{2}=1-\frac{SS_{\mathrm{err}}}{SS_{\mathrm{tot}}}\text{,}$$

where

(2) $$SS_{\mathrm{err}}=\sum_{i}{\left(y_{i}-f_{i}\right)}^{2}$$

is the sum of squared residuals with $y_{i}$ being observed and $f_{i}$ estimated output, and

(3) $$SS_{\mathrm{tot}}=\sum_{i}{\left(y_{i}-\bar{y}\right)}^{2}$$

is the total sum of squares proportional to the sample variance with $\bar{y}=\frac{1}{n}\sum_{i}^{n}y_{i}$ being the sample mean. R^2^ was used as a measure of agreement between the observed and modelled values with higher values representing a better agreement.

The second criterion – separation of the R5 and X4 phenotype vector – was used to obtain models capable of distinguishing between the two contrasting phenotypes. To measure the separation of the R5 and X4 models we used the Euclidean vector distance of the coefficient vectors of the two models. The model was selected for which the phenotype vectors of the R5 and X4 reference strains are most distant.

### Prediction of phenotype vectors

For prediction we used binary sequence encoding in which each amino acid is represented by a binary vector of the length 20 with a single value 1 at the position indicating the present amino acid. The V3 loops of the 23 tested variants in this study include 88 positions that vary among the variants. We used two methods that involve shrinkage procedures for linear regression: Ridge regression [42] and Lasso [43]. These methods were trained on the binary sequence encoding of the V3 sequences of the viruses with the respective phenotype vectors as output variables. For each position of the phenotype vector representing a separate output variable for the prediction method the penalty parameter *λ* resulting in minimal prediction error in LOOCV was chosen from a sequence of 100 values. The chosen *λ* values were used in further phenotype prediction. In addition to Ridge regression and Lasso, we tested the performance of linear regression based on a reduced number of input variables. The input variables were reduced to those showing significant (p < 0.01) Pearson correlation with any of the output variables (positions of the phenotype vector). Significance was calculated in 1000 permutation tests. The reduction procedure resulted in 26 and 17 input variables in the logarithmic and linear models of phenotype vectors, respectively.

### Optimal training set size

Each variant’s phenotype was predicted based on a sampled training set of varying size increasing from 2 to 22. Training sets of each size were sampled multiple times; the prediction error of each clone was averaged for each size of the training set. Next, a polynomial function $f(x)=ax^{b}$ termed *error function* was fitted to the relationship between the size of the training set and the prediction error. See Additional file 1: Figure S11 for examples of error functions.

## Abbreviations

HIV: Human immunodeficiency virus; MVC: Maraviroc CCR5 antagonist; AMD: AMD-3100 CXCR4 antagonist; BlaM assay: β-Lactamase virion fusion assay; V3 loop: 3^rd^ Variable region of gp120 protein; FSC: Forward scatter of flow cytometry data; SSC: Side scatter of flow cytometry data; LOOCV: Leave-one-out cross validation.

## Competing interests

We certify that there is *no competing interest* with any financial organization regarding the material discussed in the manuscript.

## Authors’ contributions

Designed and conceived experiments: HGK, TL, BM, RK, ME, KB. Performed experiments: ME, KB, SS, MA. Analyzed data: KB, ME. Discussed data: ME, KB, TL, BM, HGK, RK. Wrote the paper draft: ME, KB, all authors contributed to writing the paper and read and approved the final manuscript. Contributed patient samples: SS, RK.

## Supplementary Material

Additional file 1 “Supplementary Information” including 13 Supplementary Figures and 6 Supplementary Tables as well as additional Methods and References. Click here for file

## References

[B1] Chan DC Kim PS HIV entry and its inhibition Cell 1998 93 681 684 9630213 10.1016/s0092-8674(00)81430-0

[B2] Pierson TC Doms RW HIV-1 entry and its inhibition Curr Top Microbiol Immunol 2003 281 1 27 12932074 10.1007/978-3-642-19012-4_1

[B3] Sattentau QJ Moore JP Human immunodeficiency virus type 1 neutralization is determined by epitope exposure on the gp120 oligomer J Exp Med 1995 182 185 196 7540648 10.1084/jem.182.1.185 PMC2192089

[B4] Feng Y Broder CC Kennedy PE Berger EA HIV-1 entry cofactor: functional cDNA cloning of a seven-transmembrane, G protein-coupled receptor Science 1996 272 872 877 8629022 10.1126/science.272.5263.872

[B5] Moore JP Coreceptors: implications for HIV pathogenesis and therapy Science 1997 276 51 52 9122710 10.1126/science.276.5309.51

[B6] Trkola A Dragic T Arthos J Binley JM Olson WC Allaway GP Cheng-Mayer C Robinson J Maddon PJ Moore JP CD4-dependent, antibody-sensitive interactions between HIV-1 and its co-receptor CCR-5 Nature 1996 384 184 187 8906796 10.1038/384184a0

[B7] Berger EA Murphy PM Farber JM Chemokine receptors as HIV-1 coreceptors: Roles in viral entry, tropism, and disease Annu Rev Immunol 1999 17 657 700 10358771 10.1146/annurev.immunol.17.1.657

[B8] Miedema F Meyaard L Koot M Klein MR Roos MT Groenink M Fouchier RA Van’t Wout AB Tersmette M Schellekens PT Changing virus-host interactions in the course of HIV-1 infection Immunol Rev 1994 140 35 72 7821927 10.1111/j.1600-065x.1994.tb00864.x

[B9] Huang Y Paxton WA Wolinsky SM Neumann AU Zhang L He T Kang S Ceradini D Jin Z Yazdanbakhsh K The role of a mutant CCR5 allele in HIV-1 transmission and disease progression Nat Med 1996 2 1240 1243 8898752 10.1038/nm1196-1240

[B10] Dorr P Westby M Dobbs S Griffin P Irvine B Macartney M Mori J Rickett G Smith-Burchnell C Napier C Maraviroc (UK-427,857), a potent, orally bioavailable, and selective small-molecule inhibitor of chemokine receptor CCR5 with broad-spectrum anti-human immunodeficiency virus type 1 activity Antimicrob Agents Chemother 2005 49 4721 4732 16251317 10.1128/AAC.49.11.4721-4732.2005 PMC1280117

[B11] Fouchier RA Groenink M Kootstra NA Tersmette M Huisman HG Miedema F Schuitemaker H Phenotype-associated sequence variation in the third variable domain of the human immunodeficiency virus type 1 gp120 molecule J Virol 1992 66 3183 3187 1560543 10.1128/jvi.66.5.3183-3187.1992 PMC241084

[B12] Shioda T Levy JA Cheng-Mayer C Small amino acid changes in the V3 hypervariable region of gp120 can affect the T-cell-line and macrophage tropism of human immunodeficiency virus type 1 Proc Natl Acad Sci U S A 1992 89 9434 9438 1409653 10.1073/pnas.89.20.9434 PMC50146

[B13] Speck RF Wehrly K Platt EJ Atchison RE Charo IF Kabat D Chesebro B Goldsmith MA Selective employment of chemokine receptors as human immunodeficiency virus type 1 coreceptors determined by individual amino acids within the envelope V3 loop J Virol 1997 71 7136 7139 9261451 10.1128/jvi.71.9.7136-7139.1997 PMC192016

[B14] Raymond S Delobel P Mavigner M Ferradini L Cazabat M Souyris C Sandres-Sauné K Pasquier C Marchou B Massip P Izopet J Prediction of HIV type 1 subtype C tropism by genotypic algorithms built from subtype B viruses J Acquir Immune Defic Syndr 2010 53 167 175 19996764 10.1097/QAI.0b013e3181c8413b

[B15] Recordon-Pinson P Soulie C Flandre P Group AARS Evaluation of the genotypic prediction of HIV-1 coreceptor use versus a phenotypic assay and correlation with the virological response to maraviroc: the ANRS GenoTropism study Antimicrob Agents Chemother 2010 54 3335 3340 Aug 20530226 10.1128/AAC.00148-10 PMC2916345

[B16] Thielen A Sichtig N Kaiser R Lam J Harrigan PR Lengauer T Improved prediction of HIV-1 coreceptor usage with sequence information from the second hypervariable loop of gp120 J Infect Dis 2010 202 1435 1443 20874088 10.1086/656600

[B17] Jensen MA van 't Wout AB Predicting HIV-1 coreceptor usage with sequence analysis AIDS Rev 2003 5 104 112 12876899

[B18] Resch W Hoffman N Swanstrom R Improved success of phenotype prediction of the human immunodeficiency virus type 1 from envelope variable loop 3 sequence using neural networks Virology 2001 288 51 62 11543657 10.1006/viro.2001.1087

[B19] Pillai S Good B Richman D Corbeil J A new perspective on V3 phenotype prediction AIDS Res Hum Retroviruses 2003 19 145 149 12643277 10.1089/088922203762688658

[B20] Sing T Low AJ Beerenwinkel N Sander O Cheung PK Domingues FS Büch J Däumer M Kaiser R Lengauer T Harrigan PR Predicting HIV coreceptor usage on the basis of genetic and clinical covariates Antivir Ther 2007 12 1097 1106 18018768

[B21] Dybowski JN Heider D Hoffmann D Prediction of co-receptor usage of HIV-1 from genotype PLoS Comput Biol 2010 6 e1000743 20419152 10.1371/journal.pcbi.1000743 PMC2855328

[B22] Sander O Sing T Sommer I Low AJ Cheung PK Harrigan PR Lengauer T Domingues FS Structural descriptors of gp120 V3 loop for the prediction of HIV-1 coreceptor usage PLoS Comput Biol 2007 3 e58 17397254 10.1371/journal.pcbi.0030058 PMC1848001

[B23] Vandekerckhove LP Wensing AM Kaiser R ECGocmot: European guidelines on the clinical management of HIV-1 tropism testing Lancet Infect Dis 2011 11 394 407 May 21429803 10.1016/S1473-3099(10)70319-4

[B24] Este JA Virus entry as a target for anti-HIV intervention Curr Med Chem 2003 10 1617 1632 12871111 10.2174/0929867033457098

[B25] De Clercq E Yamamoto N Pauwels R Baba M Schols D Nakashima H Balzarini J Debyser Z Murrer BA Schwartz D Potent and selective inhibition of human immunodeficiency virus (HIV)-1 and HIV-2 replication by a class of bicyclams interacting with a viral uncoating event Proc Natl Acad Sci U S A 1992 89 5286 5290 1608936 10.1073/pnas.89.12.5286 PMC49276

[B26] Dai SJ Dou GF Qiang XH Song HF Tang ZM Liu DS Liu XW Yang LM Zheng YT Liang Q Pharmacokinetics of sifuvirtide, a novel anti-HIV-1 peptide, in monkeys and its inhibitory concentration in vitro Acta Pharmacol Sin 2005 26 1274 1280 16174446 10.1111/j.1745-7254.2005.00163.x

[B27] Este JA Telenti A HIV entry inhibitors Lancet 2007 370 81 88 17617275 10.1016/S0140-6736(07)61052-6

[B28] Westby M van der Ryst E CCR5 antagonists: host-targeted antivirals for the treatment of HIV infection Antivir Chem Chemother 2005 16 339 354 16329283 10.1177/095632020501600601

[B29] Trkola A Kuhmann SE Strizki JM Maxwell E Ketas T Morgan T Pugach P Xu S Wojcik L Tagat J HIV-1 escape from a small molecule, CCR5-specific entry inhibitor does not involve CXCR4 use Proc Natl Acad Sci U S A 2002 99 395 400 11782552 10.1073/pnas.012519099 PMC117571

[B30] Westby M Smith-Burchnell C Mori J Lewis M Mosley M Stockdale M Dorr P Ciaramella G Perros M Reduced maximal inhibition in phenotypic susceptibility assays indicates that viral strains resistant to the CCR5 antagonist maraviroc utilize inhibitor-bound receptor for entry J Virol 2007 81 2359 2371 17182681 10.1128/JVI.02006-06 PMC1865946

[B31] Salzman G Light scatter: detection and usage In Curr Protoc Cytom 2001 1.13.1 1.13.8 10.1002/0471142956.cy0113s09 18770663

[B32] Cavrois M De Noronha C Greene WC A sensitive and specific enzyme-based assay detecting HIV-1 virion fusion in primary T lymphocytes Nat Biotechnol 2002 20 1151 1154 12355096 10.1038/nbt745

[B33] Brumme C Wilkin T Su Z Schapiro J Kagan R Chapman D Heera J Valdez H Harrigan R Relative Performance of ESTA, Trofile, 454 Deep Sequencing, and “Reflex” Testing for HIV Tropism in the MOTIVATE Screening Population of Therapy- experienced Patients18th Conference on Retroviruses and Opportunistic Infections 2011 332 Abstract 666

[B34] Prosperi MC Bracciale L Fabbiani M Di Giambenedetto S Razzolini F Meini G Colafigli M Marzocchetti A Cauda R Zazzi M De Luca A Comparative determination of HIV-1 co-receptor tropism by Enhanced Sensitivity Trofile, gp120 V3-loop RNA and DNA genotyping Retrovirology 2010 30 7 56 Jun 20591141 10.1186/1742-4690-7-56 PMC2907304

[B35] Reuter S Braken P Jensen B Sierra-Aragon S Oette M Balduin M Kaiser R Häussinger D Maraviroc in treatment-experienced patients with HIV-1 infection - experience from routine clinical practice Eur J Med Res 2010 28 15 231 237 Jun 20696631 10.1186/2047-783X-15-6-231 PMC3351991

[B36] Walter H Eberle J Müller H Noah C Wolf E Stürmer M Braun P Korn K Däumer M Berg T Empfehlung zur Bestimmung des HIV-1-Korezeptor-Gebrauchs (DAIG Recommendations for the HIV-1 tropism testing) 2009 http://www.daignet.de/site-content/hiv-therapie/leitlinien-1/resolveuid/db830c8f657ca0e4735d4ff2cf665b34

[B37] Koyanagi Y Miles S Mitsuyasu R Merrill J Vinters H Chen I Dual infection of the central nervous system by AIDS viruses with distinct cellular tropisms Science 1987 15 236 819 822 May 3646751 10.1126/science.3646751

[B38] Li Y Kappes J Conway J Price R Shaw G Hahn B Molecular characterization of human immunodeficiency virus type 1 cloned directly from uncultured human brain tissue: identification of replication-competent and -defective viral genomes J Virol 1991 65 3973 3985 1830110 10.1128/jvi.65.8.3973-3985.1991 PMC248827

[B39] Ratner L Haseltine W Patarca R Livak K Starcich B Josephs S Doran E Rafalski J Whitehorn E Baumeister K Complete nucleotide sequence of the AIDS virus, HTLV-III Nature 1985 313 277 284 2578615 10.1038/313277a0

[B40] Cheng-Mayer C Levy J Distinct biological and serological properties of human immunodeficiency viruses from the brain Ann Neurol 1988 23 S58 S61 3258140 10.1002/ana.410230716

[B41] Adachi A Gendelman HE Koenig S Folks T Willey R Rabson A Martin MA Production of acquired immunodeficiency syndrome-associated retrovirus in human and nonhuman cells transfected with an infectious molecular clone J Virol 1986 59 284 291 3016298 10.1128/jvi.59.2.284-291.1986 PMC253077

[B42] Tychonoff A On the stability of inverse problems Dokl Akad Nauk SSSR 1943 39 195 198

[B43] Tibshirani R Regression shrinkage and selection via the lasso J Royal Statist Soc B 1996 58 267 288

[B44] Westby M Lewis M Whitcomb J Youle M Pozniak AL James IT Jenkins TM Perros M van der Ryst E Emergence of CXCR4-using human immunodeficiency virus type 1 (HIV-1) variants in a minority of HIV-1-infected patients following treatment with the CCR5 antagonist maraviroc is from a pretreatment CXCR4-using virus reservoir J Virol 2006 80 4909 4920 16641282 10.1128/JVI.80.10.4909-4920.2006 PMC1472081

[B45] Turville SG Cameron PU Handley A Lin G Pohlmann S Doms RW Cunningham AL Diversity of receptors binding HIV on dendritic cell subsets Nat Immunol 2002 3 975 983 12352970 10.1038/ni841

[B46] Johnston SH Lobritz MA Nguyen S Lassen K Delair S Posta F Bryson YJ Arts EJ Chou T Lee B A quantitative affinity-profiling system that reveals distinct CD4/CCR5 usage patterns among human immunodeficiency virus type 1 and simian immunodeficiency virus strains J Virol 2009 83 11016 11026 19692480 10.1128/JVI.01242-09 PMC2772777

[B47] Lengauer T Bioinformatical Assistance of Selecting Anti-HIV Therapies: Where Do We Stand? Intervirology 2012 55 108 112 22286878 10.1159/000332000

[B48] Bozek K Thielen A Sierra S Kaiser R Lengauer T V3 loop sequence space analysis suggests different evolutionary patterns of CCR5- and CXCR4-tropic HIV PLoS One 2009 4 e7387 19816596 10.1371/journal.pone.0007387 PMC2754612

[B49] Kamradt T Niese D Schneweis KE Brackmann HH Kamps B van Loo B Hammerstein U Natural history of HIV-infection in hemophiliacs: clinical, immunological, and virological findings Klin Wochenschr 1989 17 67 1033 1041 Oct 2586009 10.1007/BF01727005

[B50] Schneweis K Kleim J Bailly E Niese D Wagner N Brackmann H Graded cytopathogenicity of the human immunodeficiency virus (HIV) in the course of HIV infection Med Microbiol Immunol 1990 179 193 203 1979834 10.1007/BF00195250

[B51] Sierra S Kaiser R Lübke N Thielen A Schuelter E Heger E Däumer M Reuter S Esser S Fäetkenheuer G Prediction of HIV-1 Coreceptor Usage (Tropism) by Sequence Analysis using a Genotypic Approach J Vis Exp 2011 58 e3264 10.3791/3264 PMC3369655 22157596

[B52] Lampe M Briggs JA Endress T Glass B Riegelsberger S Kräusslich HG Lamb DC Bräuchle C Müller B Double-labelled HIV-1 particles for study of virus-cell interaction Virology 2007 30 360 92 104 Mar 17097708 10.1016/j.virol.2006.10.005

[B53] Hecky J Müller KM Structural perturbation and compensation by directed evolution at physiological temperature leads to thermostabilization of beta-lactamase Biochemistry 2005 27 44 12640 12654 Sep 16171379 10.1021/bi0501885

[B54] Münk C Brandt SM Lucero G Landau NR A dominant block to HIV-1 replication at reverse transcription in simian cells Proc Natl Acad Sci U S A 2002 15 99 13843 13848 Oct 10.1073/pnas.212400099 PMC129785 12368468

[B55] Sena-Esteves M Saeki Y Camp SM Chiocca EA Breakefield XO Single-step conversion of cells to retrovirus vector producers with herpes simplex virus-Epstein-Barr virus hybrid amplicons J Virol 1999 73 10426 10439 Dec 10559361 10.1128/jvi.73.12.10426-10439.1999 PMC113098

[B56] Means RE Matthews T Hoxie JA Malim MH Kodama T Desrosiers RC Ability of the V3 loop of simian immunodeficiency virus to serve as a target for antibody-mediated neutralization: correlation of neutralization sensitivity, growth in macrophages, and decreased dependence on CD4 J Virol 2001 75 3903 3915 Apr 11264379 10.1128/JVI.75.8.3903-3915.2001 PMC114881

[B57] Gentleman RC Carey VJ Bates DM Bolstad B Dettling M Dudoit S Ellis B Gautier L Ge Y Gentry J Bioconductor: open software development for computational biology and bioinformatics Genome Biol 2004 5 R80 15461798 10.1186/gb-2004-5-10-r80 PMC545600

