# Supplementary material for: An expanded model of HIV cell entry phenotype based on multi-parameter single-cell data
Source: Retrovirology. 2012 Jul 25;9:60. doi: 10.1186/1742-4690-9-60 (PMC3464718; doi:10.1186/1742-4690-9-60)
Supplement: Additional file 1 — “Supplementary Information” including 13 Supplementary Figures and 6 Supplementary Tables as well as additional Methods and References. [file 1742-4690-9-60-S1.doc]

*An expanded model of HIV cell entry phenotype based on multi-parameter single cell data.*

**SUPPLEMENTARY INFORMATION**

**Table of contents**

1. Cell surface expression of the HIV receptor and coreceptors on the SupT1/CCR5 cell line and on primary peripheral blood mononuclear cells (PBMCs) [3](#__RefHeading___Toc200533102)

Figure S1. Surface expression levels of HIV-1 receptor and coreceptors on SupT1/CCR5 cells as compared to PBMCs. [3](#__RefHeading___Toc200533103)

Figure S2. Range of surface expression levels of HIV-1 receptor and coreceptors on SupT1/CCR5 cells within the data set. [4](#__RefHeading___Toc200533104)

Table S1. Range of surface expression levels of HIV‑1 receptor and coreceptors on the SupT1/CCR5 cell line within the data set. [4](#__RefHeading___Toc200533105)

2. Selected sequences [5](#__RefHeading___Toc200533106)

Table S2. Characterization of V3 sequences of clones tested in this study. [5](#__RefHeading___Toc200533107)

Figure S3. Dendrogram of the tested clones and 33 V3 sequences from Los Alamos database. [6](#__RefHeading___Toc200533108)

3. Automated gating [7](#__RefHeading___Toc200533109)

Figure S4. Automated gating. [7](#__RefHeading___Toc200533110)

4. BlaM assay classification [8](#__RefHeading___Toc200533111)

Figure S5. Steps of the binary classification of cell entry based the BlaM marker. [8](#__RefHeading___Toc200533112)

Figure S6. Comparison of the exemplary results of the automated and manual gating of the BlaM assay results. [9](#__RefHeading___Toc200533113)

Figure S7. Classification based on the margin and binary methods. [10](#__RefHeading___Toc200533114)

5. Model selection [11](#__RefHeading___Toc200533115)

Table S3. Varying parameters of the classes of models tested. [11](#__RefHeading___Toc200533116)

Table S4. Quality of models based on different levels of data aggregation. [12](#__RefHeading___Toc200533117)

Table S5. Quality of models varying in the five predefined model properties (2-6, Table 4). [13](#__RefHeading___Toc200533118)

Figure S8. Evaluation of tested mathematical models based on the fit of the R5 and X4 models versus their separation. [14](#__RefHeading___Toc200533119)

6. Phenotype map based on the logarithmic model [15](#__RefHeading___Toc200533120)

Figure S9. Phenotype map based on the logarithmic model of phenotype. [15](#__RefHeading___Toc200533121)

7. Phenotype prediction [16](#__RefHeading___Toc200533122)

Figure S10. Error of the predicted phenotype vectors [16](#__RefHeading___Toc200533123)

8. Error functions [17](#__RefHeading___Toc200533124)

Figure S11. Fitted function of the prediction error [17](#__RefHeading___Toc200533125)

9. Characterization of the clones of incorrectly predicted phenotype [18](#__RefHeading___Toc200533126)

Table S6. Characterization of clones in V3 loop sequence space. [19](#__RefHeading___Toc200533127)

Figure S12. Dendrograms of three example clusters [21](#__RefHeading___Toc200533128)

10. Borderline phenotypes [22](#__RefHeading___Toc200533129)

Figure S13. Positions of borderline phenotypes on the phenotype map. [22](#__RefHeading___Toc200533130)

11. Model robustness against different levels of CCR5 expression [23](#__RefHeading___Toc200533131)

Figure S14. Model reproducibility on different CCR5 expression levels [25](#__RefHeading___Toc200533132)

REFERENCES [26](#__RefHeading___Toc200533133)

1. Cell surface expression of the HIV receptor and coreceptors on the SupT1/CCR5 cell line and on primary peripheral blood mononuclear cells (PBMCs)


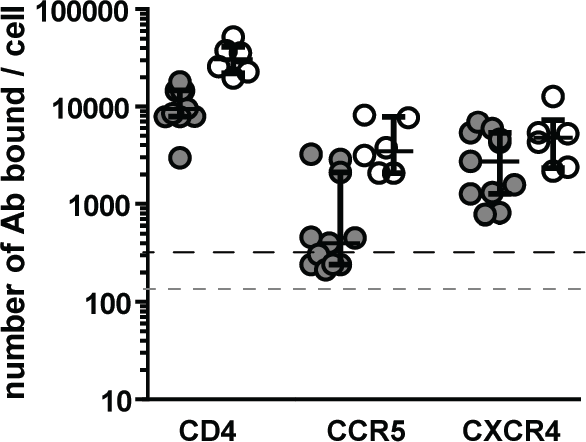


Figure S1. Surface expression levels of HIV-1 receptor and coreceptors on SupT1/CCR5 cells as compared to PBMCs.

Mean surface expression levels derived from four independent experiments are displayed in antibody molecules bound per cell for PBMCs (grey circles) and SupT1/CCR5 cells (white circles). For each experiment, PBMCs were prepared from buffy coats from two (one experiment) to three (three experiments) donors and stimulated for three to five days using IL-2 (1µg/ml) and PHA (2µg/ml). Stimulated PBMCs and SupT1/CCR5 cells were stained in parallel using PE labeled antibodies against CD4, CCR5, CXCR4, and CD19 as a negative control, respectively, and analyzed by flow cytometry. In the case of PBMCs, only staining-positive cells were taken into account for the quantitation. Raw PE-staining values were determined by quantitative flow cytometry using single stained cell populations and antibodies bound per cell were determined using the QuantiBrite PE kit (BD Biosciences) according to manufacturer’s protocol, assuming a PE:antibody ratio of 1:1. Dashed lines represent background staining of CD19-negative cell populations of PBMC (grey line) or SupT1/CCR5 cells (black line).


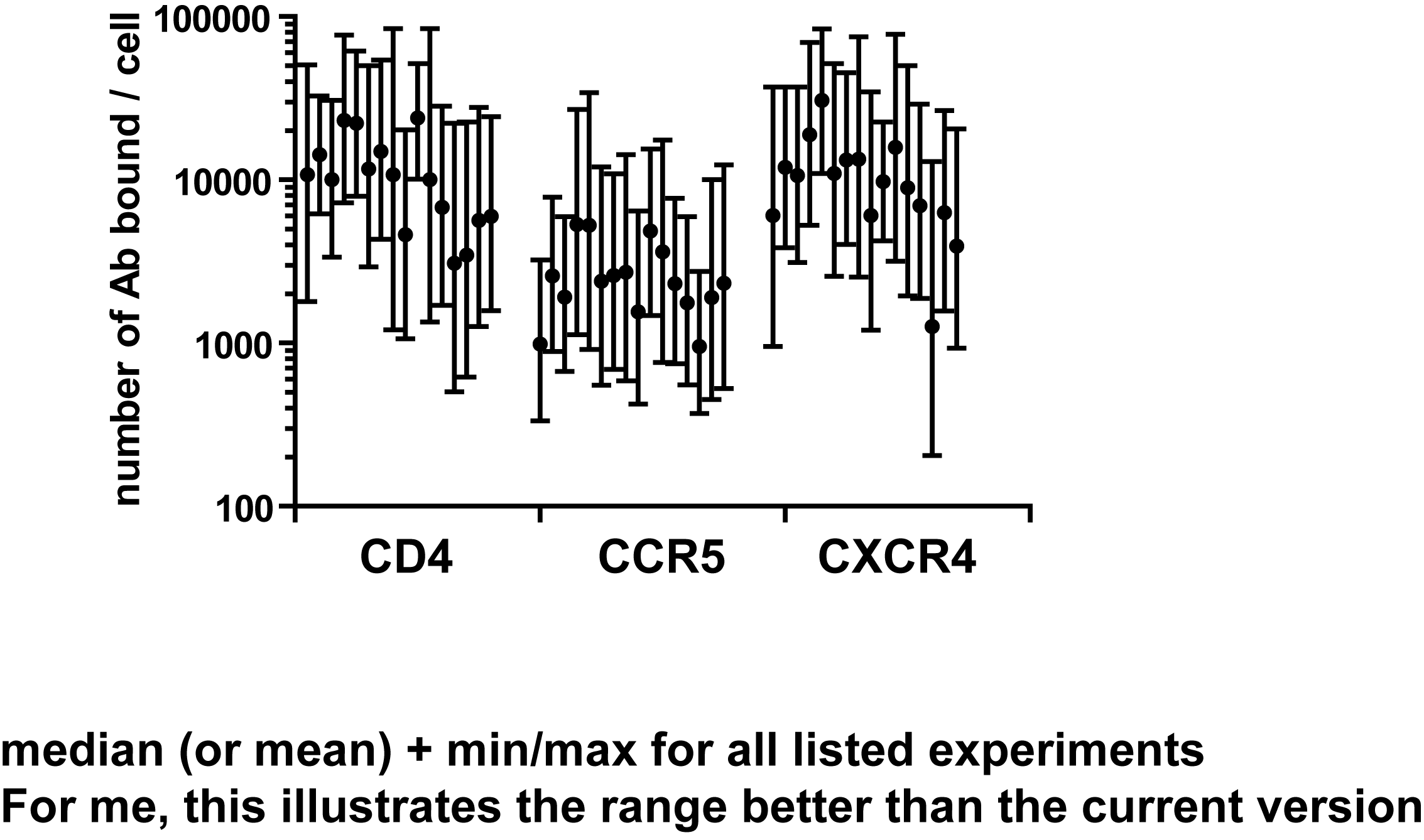


Figure S2. Range of surface expression levels of HIV-1 receptor and coreceptors on SupT1/CCR5 cells within the data set.

Mean values (dots) and minimum and maximum values (whiskers) of surface expression levels for SupT1/CCR5 cells from all 16 experiments analyzed in this study. Raw PE-staining values were determined by quantitative flow cytometry using single stained control cell populations for each experiment in parallel, numbers of antibodies bound per cell were estimated as in Figure S1.

| **molecule** | **min** | **max** |
| --- | --- | --- |
| CD4 | 3320±2974 | 45162±22517 |
| CCR5 | 691±298 | 12091±8469 |
| CXCR4 | 3024±2531 | 44379±21850 |

Table S1. Range of surface expression levels of HIV‑1 receptor and coreceptors on the SupT1/CCR5 cell line within the data set.

Mean values and standard deviation of minimum and maximum surface expression levels for SupT1/CCR5 cells from all 16 experiments analyzed in this study as determined for Figure S2.

1. Selected sequences

| **clone** | **sequence** | **phenotype** | **R5 distance** | **clustering step** | **geno2pheno**  **score (prediction)** | **WebPSSM score (prediction)** | **11/25 positions**  **(prediction)** |
| --- | --- | --- | --- | --- | --- | --- | --- |
| 220 | CTRPNNNTIKGISIGPGRAVIATRKIIGDIRQAHC | SI | 0.354 | 0.153 | 0.700 (X4) | -2.87 (X4) | GK (X4) |
| 252 | CTRPNNNTRKRITMGPGRVYYTTGDIIGDVRRAHC | SI | 0.276 | 0.102 | 0.970 (X4) | -2.58 (X4) | RD (X4) |
| 286 | CTRPHNNIKRHRIHIGPGRSFHTTKGITGNIRQAHC | SI | 0.400 | 0.280 | 0.967 (X4) | 2.63 (X4) | RG (X4) |
| 308 | CTRPNNNTIKSIRVGTGRIVYATGKIIGDIRQAHC | SI | 0.328 | 0.108 | 0.798 (X4) | 0.50 (X4) | SK (X4) |
| 315 | CTRPNNYTRKRISIGPGRAFYTTRQIIGDIRQAHC | SI | 0.302 | 0.102 | 0.974 (X4) | -3.93 (X4) | RQ (X4) |
| 376 | CTRPNNNTRKRISIGPGRSFYTTRQIIGDIRQAHC | SI | 0.277 | 0.102 | 0.914 (X4) | -5.32 (X4) | RQ (X4) |
| 381 | CTRPNNNTRKRITMGPGRVFYTTGQIIGDIRRAHC | SI | 0.276 | 0.102 | 0.950 (X4) | -2.66 (X4) | RQ (X4) |
| 391 | CTRPNNYTMRRVFIGPGRAFYAKRRIIGDIRQAHC | SI | 0.372 | 0.280 | 0.990 (X4) | -1.01 (X4) | RR (X4) |
| 468 | CTRPSNKTRTSISMGPGRAFVATRQIIGNIRQAHC | SI | 0.368 | 0.280 | 0.658 (X4) | -6.82 (R5) | SQ (R5) |
| 541 | CIRPNNNTRKGIYIGPGRAVYTTGRIIGDIRKAHC | SI | 0.301 | 0.010 | 0.856 (X4) | -6.92 (R5) | GR (X4) |
| 631 | CIRPNNNTRQRLSIGPGRAFYATRTIVGDIRQAHC | SI | 0.317 | 0.142 | 0.964 (X4) | -1.49 (X4) | RT (X4) |
| 651 | CTRPNNNTRKSVRIGPGDIFITTDIIGNIRQAHC | SI | 0.346 | 0.153 | 0.170 (R5) | -2.80 (X4) | SD (R5) |
| 685 | CTRPNNNIMRRIHIGPGRAFYATRKIIGNIRQAHC | SI | 0.344 | 0.142 | 0.983 (X4) | 0.74 (X4) | RK (X4) |
| 822 | CTRPNNNTRRSIHIAPGRAFYTTGQIIGDIRQAHC | NSI | 0.261 | 0.056 | 0.055 (R5) | -10.64 (R5) | SQ (R5) |
| 838 | CTRPNNNTRKSIHIGPGKAFYTTGEIIGDIRQAHC | NSI | 0.227 | 0.027 | 0.082 (R5) | -12.71 (R5) | SE (R5) |
| 924 | CFRPNNNTRKGIHIGPGRAFYTTGEIIGDIRRAYC | NSI | 0.275 | 0.074 | 0.316 (X4) | -8.25 (R5) | GE (R5) |
| BaL | CTRPNNNTRKSIHIGPGRALYTTGEIIGDIRQAHC | n.d. | 0.243 | 0.056 | 0.058 (R5) | -11.41 (R5) | SE (R5) |
| HxB2 | CTRPNNNTRKRIRIQRGPGRAFVTIGKIGNMRQAHC | n.d. | 0.369 | 0.153 | 1.000 (X4) | 3.27 (X4) | RK (X4) |
| JRFL | CTRPNNNTRKSIHIGPGRAFYTTGEIIGDIRQAHC | n.d. | 0.225 | 0.027 | 0.092 (R5) | -12.35 (R5) | SE (R5) |
| SF162 | CTRPNNNTRKSITIGPGRAFYATGDIIGDIRQAHC | n.d. | 0.218 | 0.049 | 0.067 (R5) | -10.50 (R5) | SD (R5) |
| YU-2 | CTRPNNNTRKSINIGPGRALYTTGEIIGDIRQAHC | n.d. | 0.248 | 0.056 | 0.040 (R5) | -12.16 (R5) | SE (R5) |
| NL4-3 | CTRPNNNTRKSIRIQRGPGRAFVTIGKIGNMRQAHC | SI | 0.359 | 0.153 | 0.928 (X4) | 0.50 (X4) | SK (X4) |
| NL4-3 R5 | CTRPNNNTRKGIHIGPGRAFYTTGEIIGDIRQAHC | n.d. | 0.230 | 0.052 | 0.104 (R5) | -12.17 (R5) | GE (R5) |

Table S2. Characterization of V3 sequences of clones tested in this study.

Column “phenotype” indicates syncytium (SI) or non-syncytium (NSI) inducing phenotype as determined on PBMC cells; n.d. not done. Columns “R5 distance” and “clustering step” indicate position of a V3 sequence in sequence space as described previously . Column “R5 distance” indicates the average distance to all R5 V3 sequences in the V3 sequence dataset studied in . Column “clustering step” indicates the step of the clustering procedure in which a sequence joins a cluster. Typical R5 sequences have been found to be more conserved than X4 sequences and therefore characterized by low values in these two columns. The three remaining columns indicate the score and phenotype predicted by three different prediction methods (geno2pheno[coreceptor] , WebPSSM and 11/25 rule ). The color indicates the predicted phenotype of each clone: blue – clearly R5, red – clearly X4, magenta – ambiguous.

| 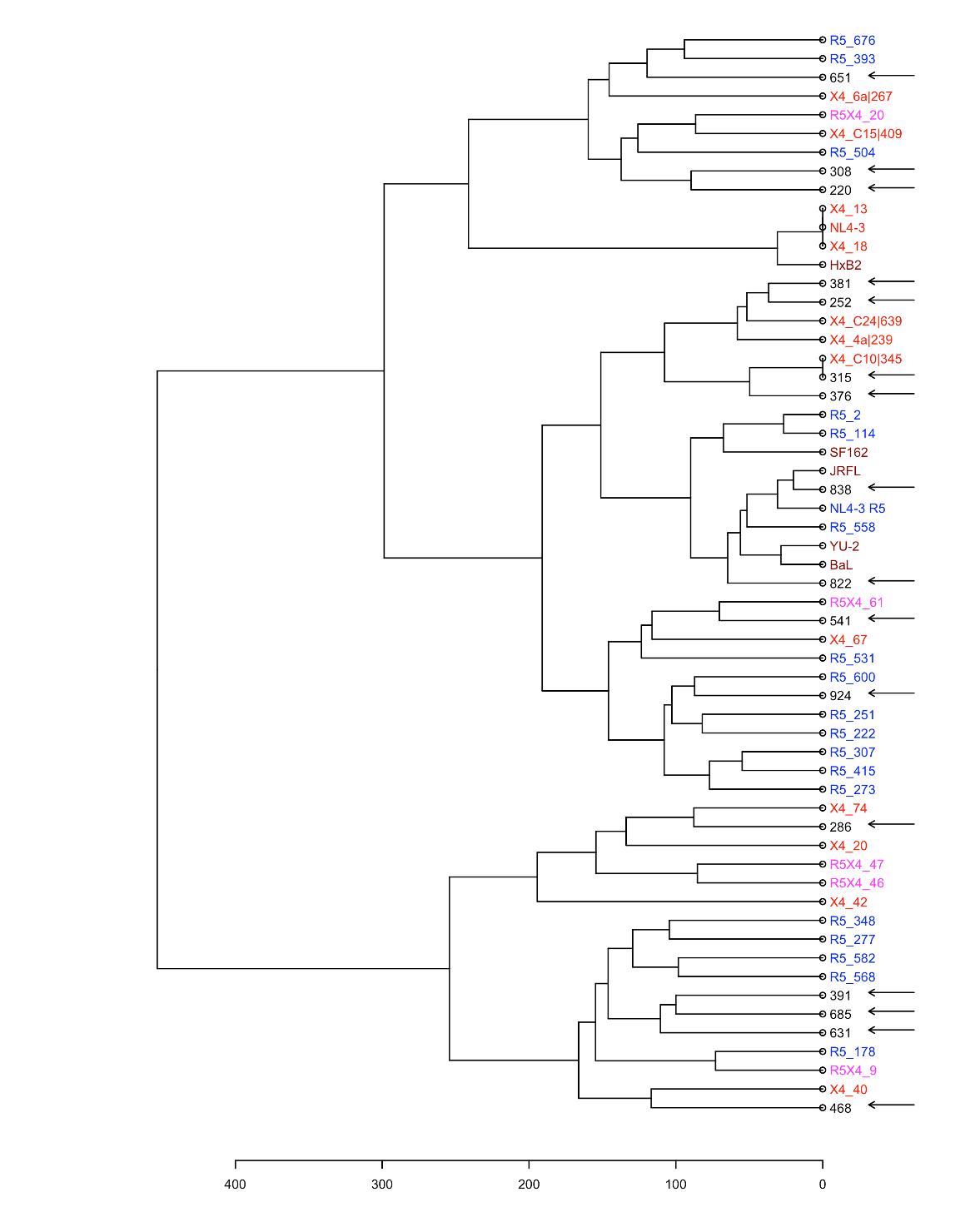 |
| --- |

Figure S3. Dendrogram of the tested clones and 33 V3 sequences from Los Alamos database.

The sequences of the patient samples and the lab-adapted clone sequences were incorporated into a set of 793 V3 sequences of annotated tropism from the Los Alamos database (http://www.hiv.lanl.gov/). The dataset was hierarchically clustered and the optimal clustering of the sequences was chosen based on the silhouette value yielding 50 clusters. 11 of the clusters contained clones tested in this study. Three sequences from each of the 11 clusters were sampled randomly. The sampled database sequences are plotted together with the sequences of the selected clones. R5 sequences are colored in blue, X4 in red, dual-tropic in magenta. Clones tested in this study are indicated with arrows. Labels of clones from the Bonn cohort are colored in black, labels of lab-adapted clones in dark red.

1. Automated gating

| 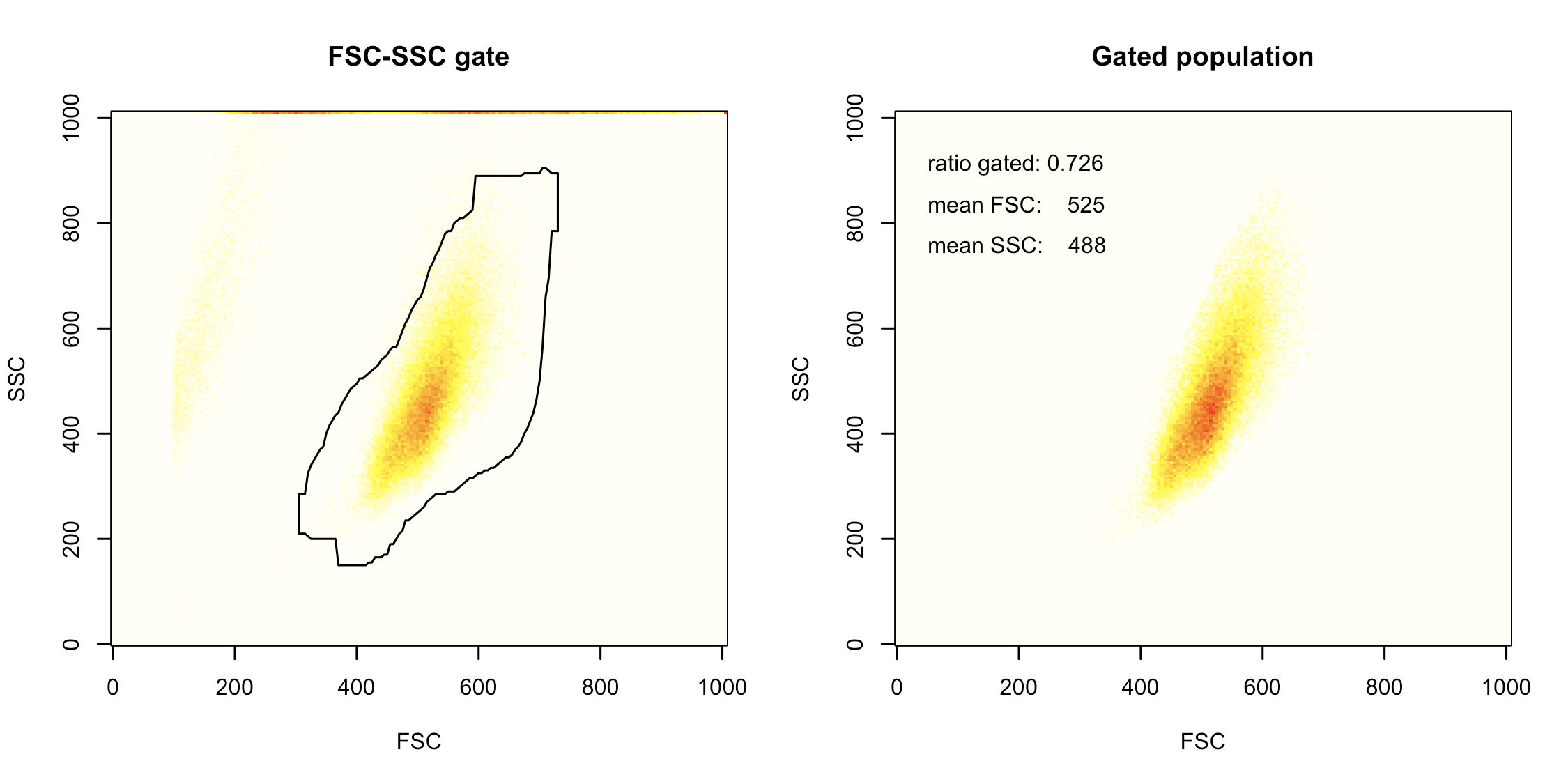 |
| --- |

Figure S4. Automated gating.

The left panel shows a heat map of the cell density showing specific FCS and SSC values before gating. The calculated gate is traced with a black line. A heat map of the gated cell population is shown on the right panel. The cell population with small FSC and high SSC values as compared to the main population is removed in this example.

1. BlaM assay classification

| 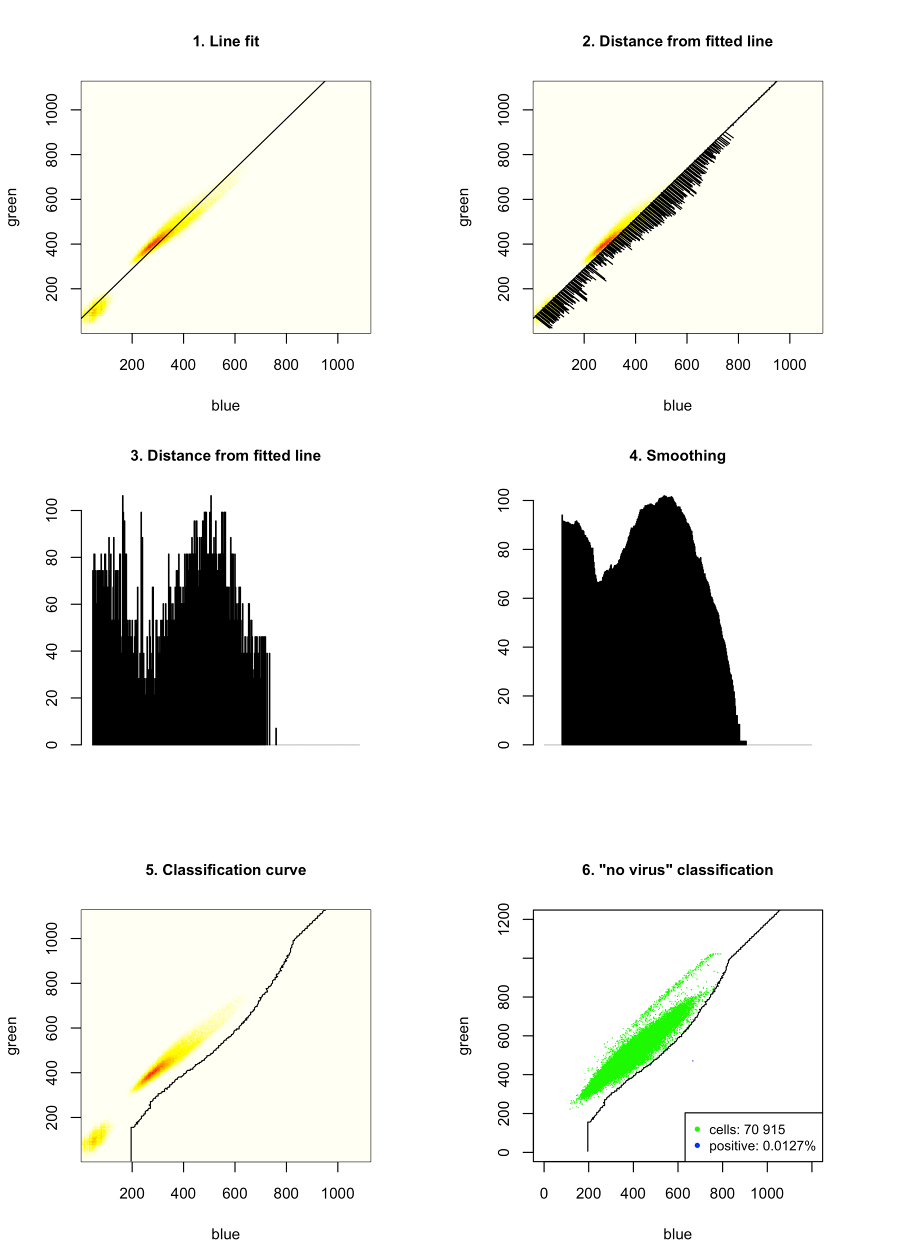 |
| --- |

Figure S5. Steps of the binary classification of cell entry based the BlaM marker.

(1) Heat map of the distribution of uninfected SupT1/CCR5 cells in the control measurement showing given combinations of blue and green fluorescence intensities. The line represents the linear function fitted to the values of the intensity of the blue and green signal. (2) Identification of data points most distant from the fitted line towards higher ratio of blue to green signal intensity. (3) Distances of these points from the fitted line are measured and (4) smoothed in a sliding window approach. (5) Smoothed curve mapped back onto the blue and green fluorescence heatmap defines the classification curve. (6) Plot of blue and green signal values of the cells of the control measurement of uninfected cells. The classification curve determined in the previous steps is shown in black, the number of cells and the percentage of control cells outside the classification curve are indicated in the legend.

| 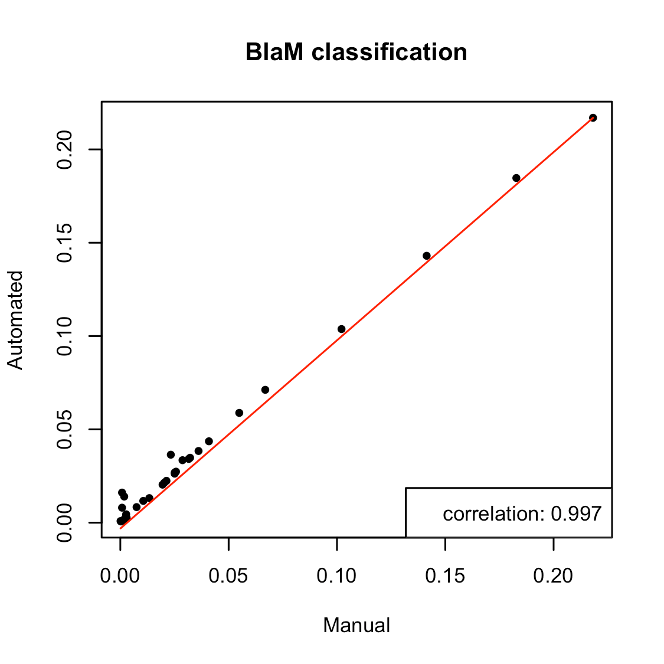 |
| --- |

Figure S6. Comparison of the exemplary results of the automated and manual gating of the BlaM assay results.

Thirty-one datasets obtained from FACS measurements of exemplary virus entry experiments were classified both manually and using the described automated approach. The plot shows the correlation between the percentage of entry positive cells determined manually or using the automated approach, respectively.

**Margin classification.** Comparison of the results of the automated and manual classification methods indicated their lower agreement for samples with high cell numbers close to the decision line. In order to account for potential errors of the automated method for these samples, we devised an alternative classification method that instead of a cutoff curve calculates a region delimited by two parallel margins surrounding the decision curve of the binary method (Figure S5, upper panel). Cells located within the region delineated by these two curves are assigned a value between 0 (entry negative) and 1 (entry positive) that reflects the probability that a given cell belongs to each of the two classes. The probability estimate is calculated as proportional to the distance of a cell from the upper boundary of the region (lower blue-to-green ratio) with probability 0 at this boundary and 1 at the bottom boundary (higher blue-to-green ratio).

| **A**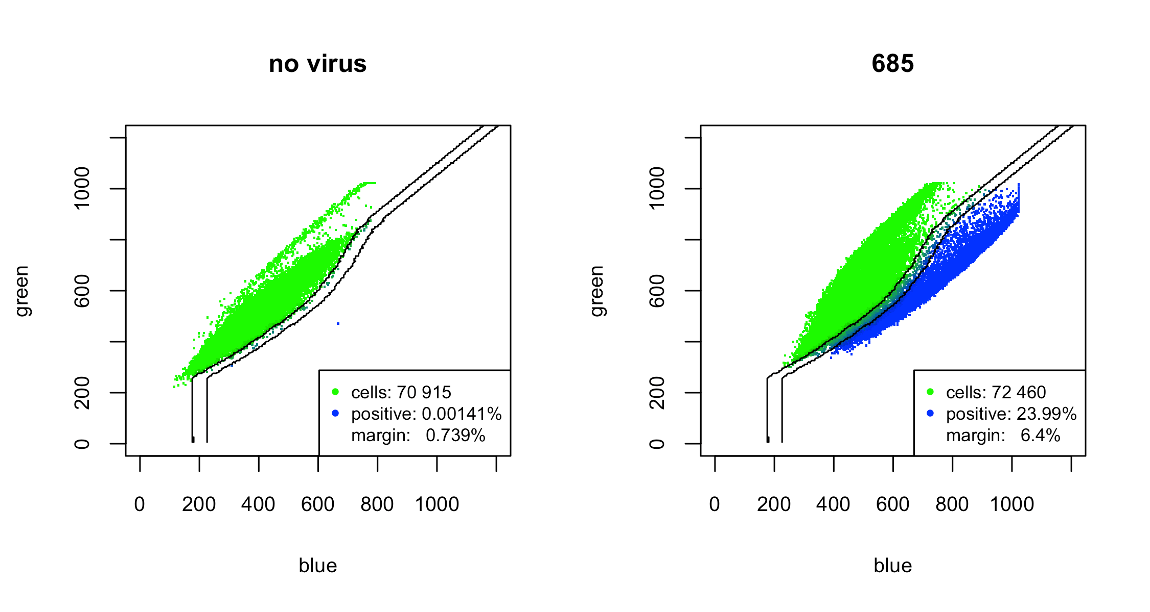 |
| --- |
| **B**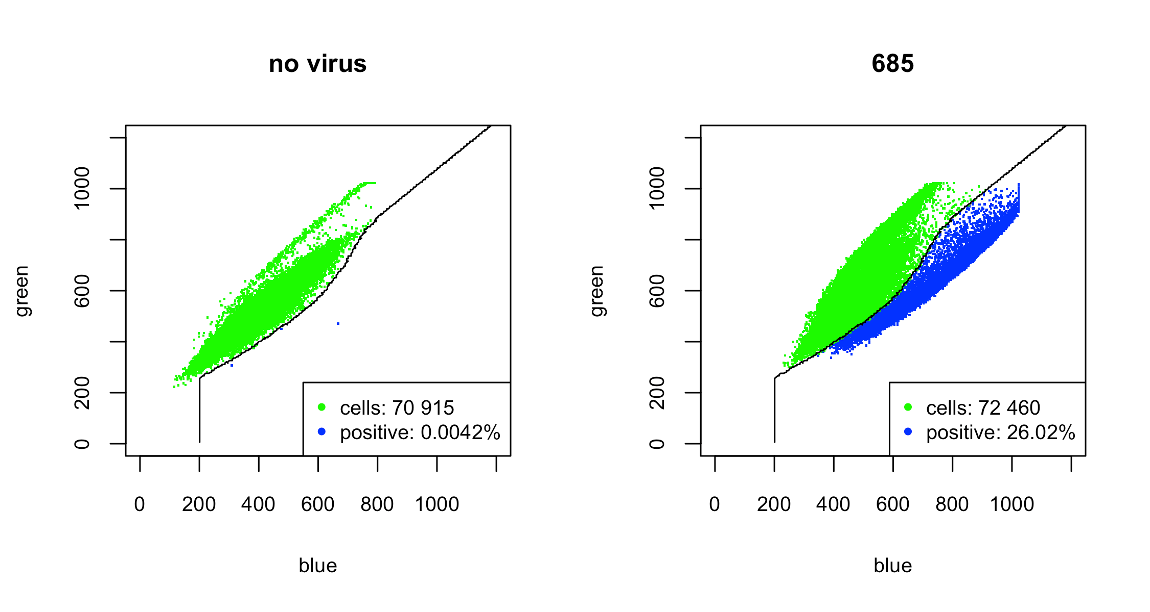 |

Figure S7. Classification based on the margin and binary methods.

(A) Two black lines trace the margin between entry negative (green) and entry positive (blue) cells. Cells located within the margin are colored according to the assigned probability with a green shade representing values closer to 0 and blue closer to 1. (B) Binary classification of these two examples. Classification of a virus measurement (right panel) is based on the classification curve established based on the no virus control (left panel). Cells classified as entry positive are represented by blue dots, entry negative cells by green dots. The percentage of entry positive cells is indicated in the legend.

1. Model selection

Models varying in different properties were trained and evaluated on the entire set of entry efficiency data of the reference X4 and R5 clones. Since the regression models constructed in this part of the study were used as multivariate descriptors of virus phenotype and not for its prediction, the model fit to the entire cell entry data, rather than its predictive power in a cross validation setting was used as a criterion for model selection.

We limited the large number of possible models that can be defined on the basis of five input variables to those varying in six different parameters listed in Table S3, numbered from 1 to 6 and discussed below.

|  | **model property** | **values tested** |  |
| --- | --- | --- | --- |
| 1 | aggregation level | 5 / 10 / 20 / 50 |  |
| 2 | response variable | binary / margin |  |
| 3 | underrepresented grid cells | 0 / 10 / 20 |  |
| 4 | drug concentration scale | linear / logarithmic |  |
| 5 | drug-coreceptor combination | yes / no |  |
| 6 | drug-drug combination | yes / no |  |

Table S3. Varying parameters of the classes of models tested.

The aggregation level (1) corresponds to the number of bins into which the coreceptor and receptor expression levels are partitioned to produce a data grid. The tested bin numbers are 5, 10, 20 and 50. The response variable (2) is based on the classification method – binary or margin (see description in the main text and above). The “underrepresented grid cells” property (3) defines the cutoff for filtering out the parts of the data grid that contain numbers of single-cell measurements below a given value. The basis for introducing this filter was the observation that the distribution of residuals of the models exhibited a tail of high residual values supposedly due to the high variance of entry efficiencies estimated from a low number of single cell measurements in certain parts of the data grid. Elimination of parts of the data grid with a small number of cells resulted in a normal distribution of residuals. “Drug concentration scale” (4) involves two scales of the MVC and AMD concentration parameters – linear and logarithmic. As more measurements were performed with low drug concentrations the logarithmic scale produced a more balanced coverage of the range of the values of MVC and AMD variables which might result in a better model fit. The properties “drug-coreceptor combination” (5) and “drug-drug combination” (6) describe models in which, in addition to the individual effects of the coreceptor expression levels and drug concentrations on the response, their combined effect is also tested. These models include the effect of all combinations of values of a coreceptor expression level and the corresponding drug concentration (5), i.e., combinations of MVC concentration with CCR5 expression and AMD concentration with CXCR4 expression, and of the combinations of values of the two drugs (6). The combination of the varied model properties resulted in 192 models tested, 48 for each aggregation level.

The significance of model variable coefficients was also inspected. Models showing insignificant variable coefficients (p > 0.05) were reduced by removing the respective input variables and recalculated. We used the analysis of variance (ANOVA) method on nested models excluding each of the input variables separately. The F-test was used to assess the significance of a coefficient in the model before reducing as compared to the respective nested model.

Strong data aggregation resulted in a higher number of reduced models – among models based on data aggregated to 5 bins 64.6% were reduced, among those based on data aggregated to 50 bins 25.0% were reduced (Table S4). Notably, only the coefficients of combined variables appeared insignificant, coefficients of coreceptor and receptor expression levels as well as drug concentrations were significant in all tested models. Table S4 lists the quality of models based on different levels of aggregation. It appears that aggregation 50 resulted in models of a markedly lower fit to the data (R^2^ ~ 0.10 on average) as compared to stronger data aggregations 5, 10 and 20 (R^2^ ~ 0.47 on average). In the following analysis only models based on the aggregations 5, 10 and 20 were considered.

| **aggregation** | **reduced models [%]** | **max R^2^ (mean)** | | **max R5-X4 distance (mean)** |
| --- | --- | --- | --- | --- |
|  |  | **R5 model** | **X4 model** |  |
| 5 | 64.6 | 0.726 (0.516) | 0.622 (0.521) | 1.715 (1.585) |
| 10 | 58.3 | 0.711 (0.467) | 0.632 (0.474) | 1.781 (1.594) |
| 20 | 33.3 | 0.676 (0.406) | 0.615 (0.426) | 1.943 (1.623) |
| 50 | 25.0 | 0.131 (0.102) | 0.126 (0.108) | 1.805 (1.636) |

Table S4. Quality of models based on different levels of data aggregation.

Column “reduced models” indicates the percentage of models that were reduced by removing the variables with insignificant coefficients. Model quality is based on the model fit to the data (R2) and separation of the R5 and X4 models (R5-X4 Euclidean distance of the parameter coefficient vectors).

Table S5 lists the quality of models grouped according the five remaining model properties. Among them, excluding underrepresented data grid cells (3) resulted in an increase of the model fit and the R5-X4 model separation. Excluding grid bins that contain below 20 data points resulted in mean R^2^ ~ 0.57 and mean R5-X4 model distance ~1.62 as compared to the mean R^2^ ~ 0.30 and the mean R5-X4 model distance ~1.59 of the models based on data where only grid bins containing below 5 data points were filtered. In the data aggregation to 5, 10 and 20 bins the filters removed less than <1%, 2-6.5% and 25-50% single-cell measurements respectively. Also the logarithmic drug concentration scale (4) resulted in models with a higher fit to the data (mean R^2^ ~ 0.54) than the linear scale (mean R^2^ ~ 0.39). For the remaining model properties (2, 5, 6) a relationship of the properties with the model fit and the R5-X4 model separation was not observed.

|  |  |  | **max R^2^ (mean)** | | **max R5-X4 distance (mean)** |
| --- | --- | --- | --- | --- | --- |
|  |  |  | **R5 model** | **X4 model** |  |
| 2 | response | binary | 0.716 (0.456) | 0.652 (0.464) | 1.943 (1.614) |
|  |  | margin | 0.726 (0.469) | 0.662 (0.484) | 1.904 (1.587) |
| 3 | under-represented grid cells | 5 | 0.468 (0.281) | 0.491 (0.320) | 1.655 (1.519) |
|  |  | 10 | 0.690 (0.530) | 0.643 (0.527) | 1.655 (1.519) |
|  |  | 20 | 0.726 (0.577) | 0.662 (0.574) | 1.943 (1.659) |
| 4 | drug concent-ration scale | linear | 0.480 (0.369) | 0.563 (0.425) | 1.943 (1.660) |
|  |  | logarithmic | 0.726 (0.557) | 0.662 (0.523) | 1.737 (1.541) |
| 5 | drug-coreceptor combination | no | 0.722 (0.462) | 0.661 (0.473) | 1.943 (1.621) |
|  |  | yes | 0.726 (0.463) | 0.662 (0.475) | 1.781 (1.580) |
| 6 | drug-drug combination | no | 0.723 (0.462) | 0.662 (0.473) | 1.885 (1.579) |
|  |  | yes | 0.726 (0.463) | 0.662 (0.475) | 1.943 (1.622) |

Table S5. Quality of models varying in the five predefined model properties (2-6, Table 4).

Models are based on the data aggregation to 5, 10 and 20 segments and those not satisfying the parameter coefficient criterion are reduced accordingly. Maximum and mean model fit to the data (“R2”) and the best and mean separation of the R5 and X4 models (R5-X4 distance) are shown for models grouped according to each of the properties.

We next inspected the relationship between the model fit to the data and the R5-X4 model separation (Figure S8). We selected two models for further inspection – one offering the best R5-X4 model separation and another one exhibiting a better model fit at the cost of a lower model separation. The two models (indicated with arrows in Figure S8) differ in model property “drug concentration scale” (4) with the logarithmic scale showing a better model fit (R^2^ ~ 0.62 vs. 0.49) and the linear scale a better model separation (R5-X4 model distance of ~ 1.94 vs. 1.74). These models were termed *logarithmic model* and *linear model* respectively. The remaining properties are shared by both models and amount to:

1. aggregation level: 20

2. response variable: binary method

3. underrepresented data grid cells: 20

5. drug-coreceptor combination: yes

6. drug-coreceptor combination: yes

| 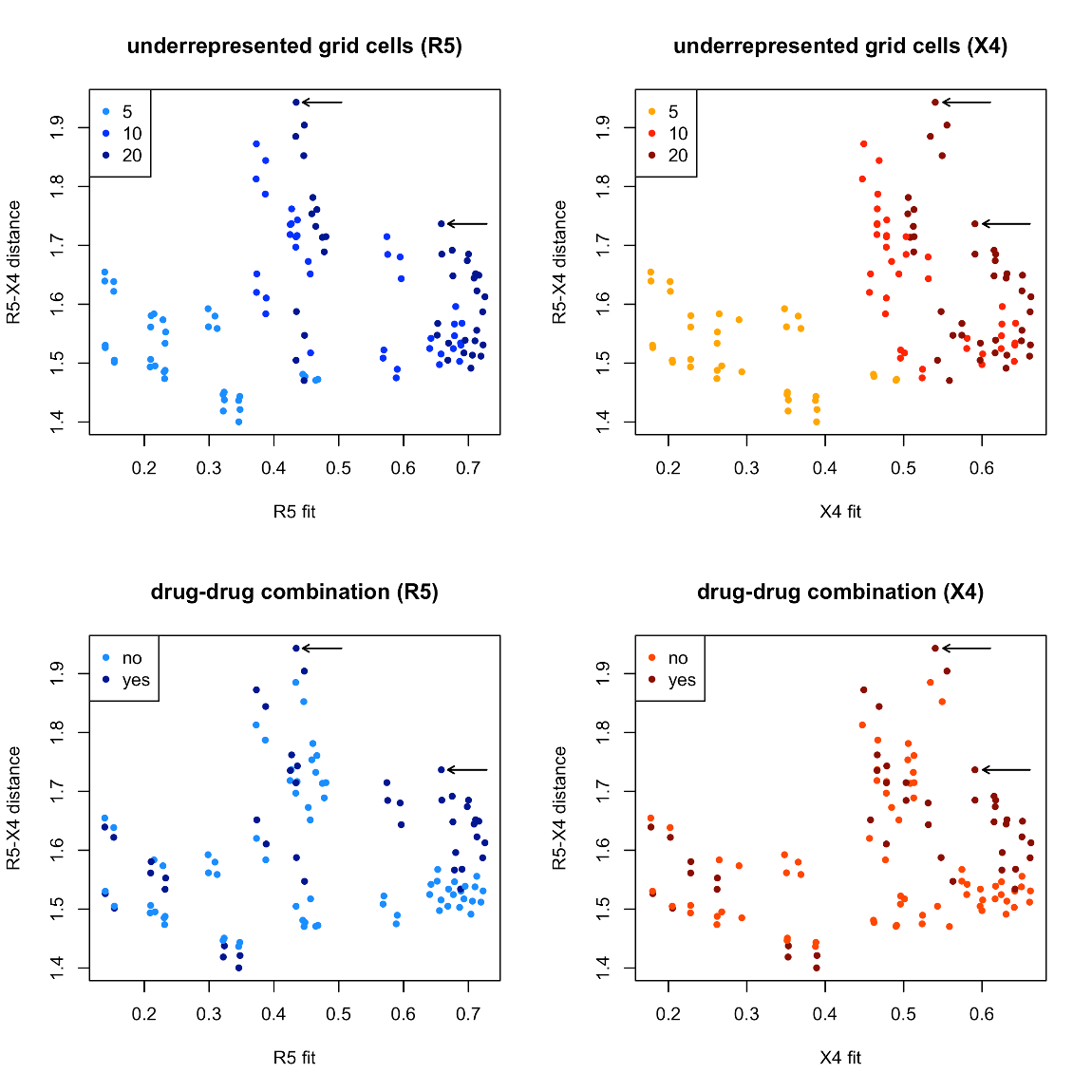 |
| --- |

Figure S8. Evaluation of tested mathematical models based on the fit of the R5 and X4 models versus their separation.

Dots represent the tested models. Left panels show R5 models and right hand side panels X4 models, respectively. Color shades correspond to the values of two properties of the models: underrepresented grid cells (property 3, upper panels) and drug-drug combination (property 6, bottom panels). The optimal model is defined as the one offering the highest R5-X4 distance with the best fit to the data and would be located in the top-right corners of both plots. The property “underrepresented grid cells” (upper panels) is displayed as an example property that shows a clear relationship with the model fit to the data. In contrast the property “drug-drug combination” (bottom panel) is an example of a property evenly distributed among models of different fit and separation therefore having no effect on the model quality. Selected models – logarithmic (better fit) and linear (better separation) – are indicated with arrows.

1. Phenotype map based on the logarithmic model

| 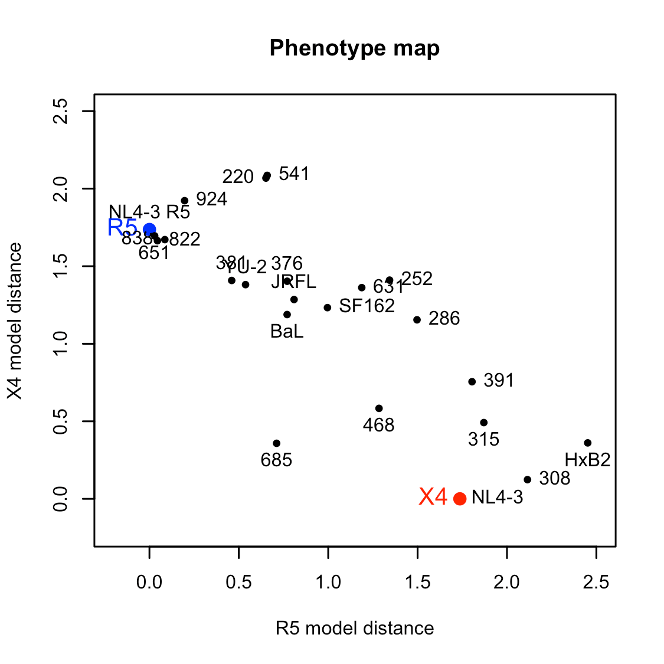 |
| --- |

Figure S9. Phenotype map based on the logarithmic model of phenotype.

The clones show a similar distribution as on the map based on the linear model (Figure 4 in the main text) with the dual-tropic clones 685, 252 and 631 located in-between clones of R5 and X4 phenotype.

1. Phenotype prediction

The binary encoding of the amino acid sequences of the V3 loops of the 23 tested clones includes 88 positions that show variation among the clones. To predict the phenotype vector from sequence based on a low number of observations, we tested two methods involving regularization procedures (Ridge regression and Lasso ) as well as linear regression based on a reduced number of input variables.

| **Linear phenotype model** |
| --- |
| 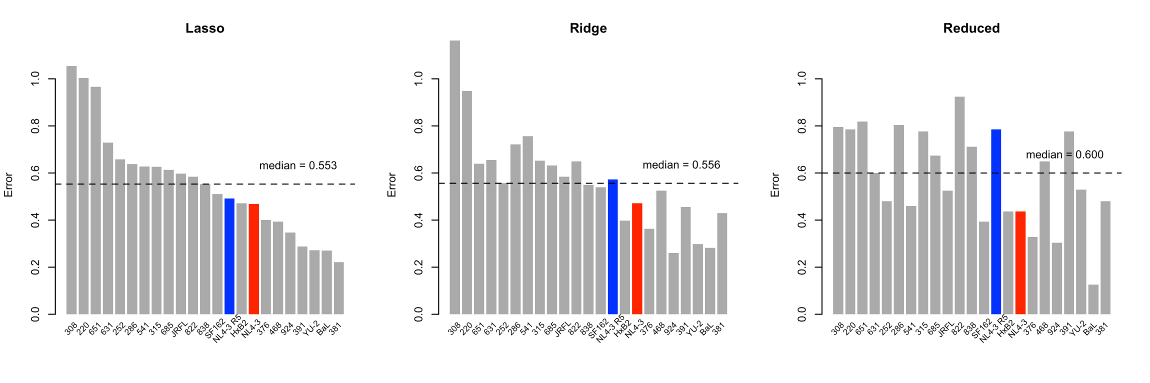 |
| **Logarithmic phenotype model** |
| 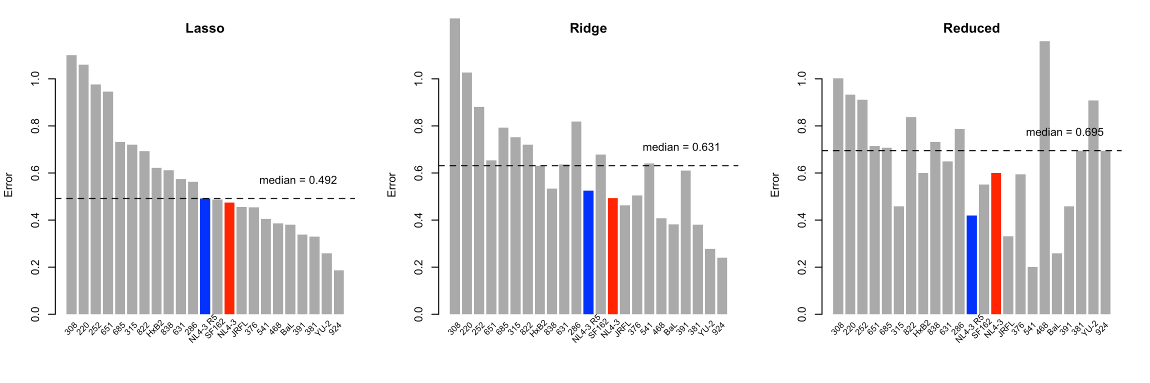 |

Figure S10. Error of the predicted phenotype vectors

Shown are prediction errors of three models – Lasso (left-hand plots), Ridge (middle plots) and reduced linear model (right-hand plots). Predictions are calculated in a LOOCV setting, error is estimated as Euclidean distance between the predicted and observed phenotype vectors. Bars are ordered according to the error of the Lasso model (left-hand plots). Blue and red bars depict R5 and red X4 reference clones respectively. Dashed lines depict the median of error of each method with its value indicated above the line. Top panels present prediction errors of the phenotypes based on linear models, bottom panels – of the logarithmic models. The R5 reference clone is colored in blue, the X4 reference clone in red.

Figure S10 shows prediction errors of the three models on the tested clones. The reduced linear model showed the lowest accuracy, presumably due to the number of input variables exceeding the number of observations in this model. Between ridge and lasso prediction models similar relative prediction errors among clones were observed. Clones 308, 220 and 651 showed high prediction errors in the models produced by Lasso method both based on linear and logarithmic phenotype models.

1. Error functions

| 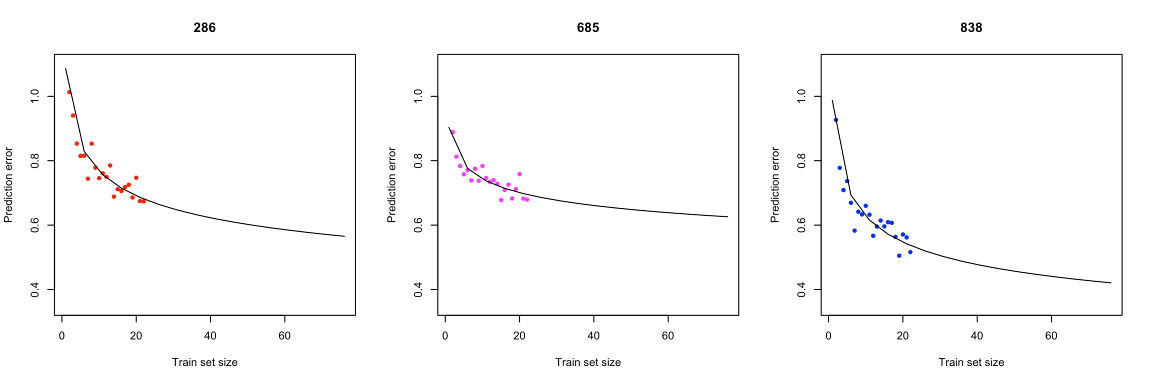 |
| --- |

Figure S11. Fitted function of the prediction error

Fitted error functions against the size of training set for three example clones: an X4 clone (286, left-hand panel), ambiguous clone (685, middle panel) and an R5 clone (838, right-hand panel). Dots represent the averaged prediction error obtained for sampled training sets of respective sizes. Black lines represent the fitted function plotted in the range (0, 75] of the training set sizes.

1. Characterization of the clones of incorrectly predicted phenotype

Three clones in our dataset (220, 308 and 651) showed high prediction errors and a lack of decrease in the prediction error with an increasing size of the training set. In order to provide a confidence measure for the clone phenotype prediction using our method, we elaborated a quantitative description of these clones. The analysis was based on two factors that might affect the accuracy of clone phenotype prediction: presence of a genetically similar V3 loop in the training set, and the position of the clone in the V3 loop sequence space. Genetically similar clones in the training set might provide information on the sequence features that are determinant for the phenotype of a given clone. The location of a clone sequence in the V3 loop sequence space indicates either its close affinity to sequences of a particular tropism or its location at the boundaries between regions of different tropism where sequence features informative of a specific phenotype might prove difficult to interpret.

Table S6 lists characteristics of the sequences of the tested clones grouped according to sequence clusters as pictured on Figure S3. Column “closest clone” indicates the genetically most closely related clone among all clones in the training set and its distance, expressed as the percentage of pairs of V3 sequences in sequence space located at smaller distances from each other than the distance between the given two clones. This measure indicates how distant the given clone is from the closest clone in the training set, as compared to the overall spread of V3 sequences in sequence space. Low values in this column indicate clones for which a genetically similar clone can be found in the training set; high values indicate clones of low genetic similarity to other clones in the training set. The following columns of Table S6 depict clone position in their clusters and the respective cluster composition. We define the *cluster center* to be the sequence of a minimal mean distance to other sequences within the cluster and *cluster radius* as the maximal distance of a cluster sequence to the cluster center. Column “distance to cluster center” indicates the distances of the clone sequences to the center of cluster they belong to relative to the cluster radius. Distance 0 is characteristic of a cluster center, distance 1 of the most distant sequence of a cluster, termed here *cluster boundary*.

To depict the composition of a cluster we introduced the cluster score:

$$c_{i}=\frac{(r5_{i}-x4_{i})}{(r5_{i}+x4_{i}+r5x4_{i})}$$

where

$$r5_{i},x4_{i},r5x4_{i}$$

are the numbers of R5, X4 and dual-tropic sequences, respectively, in cluster *i.* Cluster scores close to 0 are characteristic of mixed and dual-tropic clusters, close to -1 of predominantly X4 clusters, close to 1 of predominantly R5 clusters.

| **cluster** | | | | **clone** | **closest clone (smaller dist.[%])** | **distance to cluster center** |
| --- | --- | --- | --- | --- | --- | --- |
| **nb** | **score** | **R5s** | **X4s** |
| 1 | 0.95 | 96 | 1 | 838  JRFL  SF162  BaL  YU-2  NL4-3 R5 | JRFL (0.3)  838 (0.3)  JRFL (3.1)  JRFL (0.5)  BaL (0.7)  JRFL (0.3) | 0.44  0.32  0.40  0.56  0.84  0.48 |
| 2 | 0.93 | 139 | 1 | 822 | JRFL (1.2) | 0.63 |
| 3 | 0.92 | 44 | 0 | 924 | NL4-3 R5 (2.0) | 0.69 |
| 4 | 0.63 | 11 | 1 | 631 | 685 (24.3) | 0.80 |
| 5 | -0.13 | 9 | 12 | 541 | NL4-3 R5 (7.8) | 0.14 |
| 6 | -0.10 | 7 | 9 | **220**  **651** | 376 (24.3)  SF162 (30.3) | 0.53  0.67 |
| 7 | -0.36 | 1 | 5 | 252  315  376  381 | 381 (0.9)  376 (2.0)  315 (2.0)  252 (0.9) | 0.26  0.91  0.74  0 |
| 8 | -0.17 | 4 | 6 | 391  468  685 | 685 (18.6)  631 (37.4)  391 (18.6) | 0.51  0.81  0 |
| 9 | 0.79 | 12 | 1 | **308** | BaL (22.7) | 1.00 |
| 10 | -1.00 | 0 | 2 | HxB2  NL4-3 | NL4-3 (0.5)  HxB2 (0.5) | 0  1.00 |
| 11 | -0.75 | 0 | 3 | 286 | 685 (23.4) | 0 |

Table S6. Characterization of clones in V3 loop sequence space.

Clones are grouped according to the sequence cluster to which they belong as described in the main text. Cluster number, score, numbers of R5 and X4 sequences and clones pertaining to each cluster are indicated in columns 1-5. The closest clone among the tested clones is indicated in the column “closest clone” with the percentage of pairs of V3 sequences in sequence space located at a smaller distance from each other listed in parentheses. Distance to the cluster center and cluster score are measured as described in the text above. Three clones characterized by this analysis are highlighted in red.

Clusters 1-4, 7, 9-11 represent relatively uniform X4 or R5 clusters as reflected by the value of the cluster score close to -1 and 1, respectively. It can be assumed that clones that belong to these clusters are located in parts of sequence space predominantly occupied by sequences of the same tropism with which they share the sequence characteristics. Clone 308 belonging to cluster 9 represents the most distant sequence from the cluster center located on the cluster boundary (distance to the cluster center = 1). This cluster is predominantly R5, however, the closest neighbor of 308 in this cluster is the only X4 sequence in this cluster, suggesting that the ambiguous sequence characteristics of this clone comprise both R5 and X4 sequence characteristics (Figure S12, middle panel). In cluster 10, clone HxB2 is also located on the cluster boundary, however, unlike clone 308, this clone is closely related to another clone in this cluster (NL4-3) that is informative for its phenotype prediction.

Among the remaining clusters, cluster 6 shows the score closest to 0 (score = ‑0.1) of the analyzed clusters, indicating its mixed composition. This cluster is also sparse as reflected by long branches of the respective tree (Figure S12, left panel). Two clones pertaining to this cluster – 220 and 651 – are relatively distant from other tested clones, showing distances from the upper 0.20 quantile of the distance distribution among all V3 sequences used, which underlies the difficulty of their phenotype prediction. Clone 541 belongs to another mixed cluster 5. However the sequence similarity of this clone to the clone NL4-3 R5 appears to be sufficient for the accurate prediction of its phenotype. Similarly, clones 391 and 685 contained in a relatively mixed cluster 8 (score = ‑0.17) are each other’s closest neighbors among the tested clones, which might contribute to the accurate prediction of their phenotypes.

| Cluster 6 | Cluster 9 | Cluster 7 |
| --- | --- | --- |
| 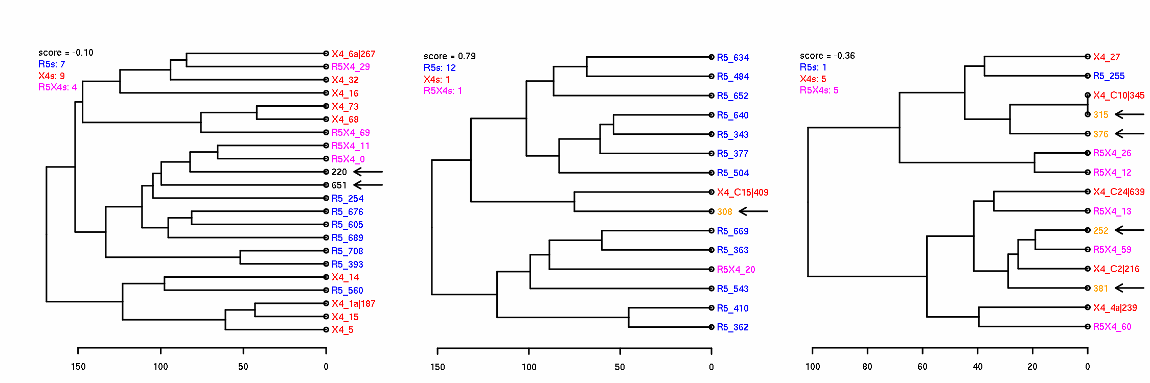 | | |

Figure S12. Dendrograms of three example clusters

Dendrograms are based on the clustering described in the main text. Cluster 6 (left-hand panel) is mixed and sparse as reflected by the equal proportion of R5 and X4 sequences in this cluster and long tree branches. Cluster 9 (middle panel) is a uniform R5 cluster with clone 308 located on its boundaries. The prediction error of the clones 220, 651 and 308 belonging to these two clusters does not show improvement with the increasing size of the training set. Cluster 7 (right-hand panel) is predominantly X4 and contains four closely related clones from the train set. Clones pertaining to this cluster show an increase in phenotype prediction accuracy with the increasing size of the train set.

Overall, this analysis indicates that a sequence location on a tropism boundary (clone 308) or a location in a mixed cluster with no close neighbors among the tested clones (clones 220, 651) incurs difficulties for the phenotype prediction. With a training set comprising more sequences that populate a larger number of clusters, a systematic approach to establishing a confidence score for the phenotype prediction will be possible. Based on the current clone set, we propose two rules for identifying sequences prone to erroneous phenotype prediction:

- sequences located on the boundaries of clusters not containing other clones from the training set, or
- sequences belonging to mixed clusters (absolute cluster score < 0.15) and distant from other tested clones (distance greater than at least 20% of distances in sequence space).

These two rules characterize clones 308, 220 and 651 in our dataset and 68 out of 880 sequences from the V3 sequence dataset used for this analysis. Experimental testing of the phenotype mediated by these sequences is of high interest for further development of our approach.

1. Borderline phenotypes

| 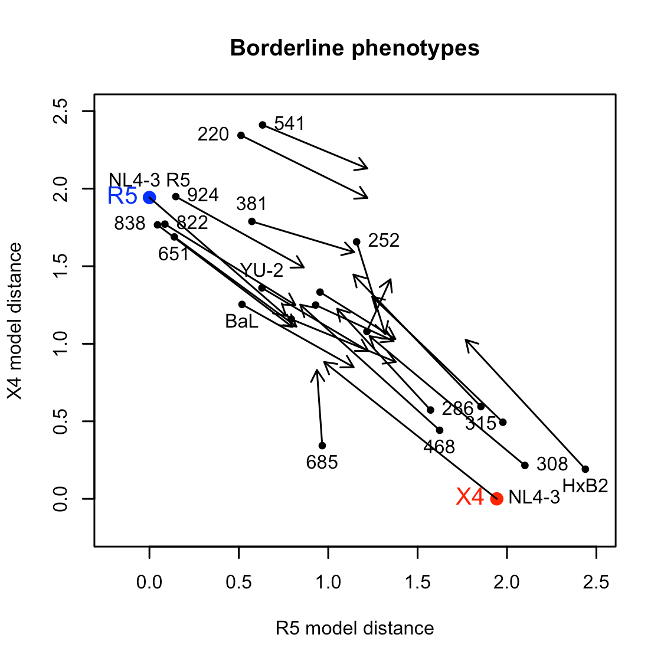 |
| --- |

Figure S13. Positions of borderline phenotypes on the phenotype map.

Arrows connect the phenotypes of the observed clones with the respective borderline phenotypes. Borderline phenotypes represent the minimal change in the observed phenotype vector that would result in a phenotype misclassification.

1. Model robustness against different levels of CCR5 expression

We did not consider primary cells suitable for the development of the presented model due to the high degree of donor variability. The SupT1/CCR5 cell line used in our study exhibits higher average CCR5 surface expression levels than primary T cells, however, CCR5 surface levels are overlapping between these cell populations. We tested the validity of our results in dependence of the average coreceptor level displayed on the cells. For this, we stratified the SupT1/CCR5 datasets used for model development according to the CCR5 surface levels on individual cells. Cell populations were separated in five quintiles spanning consecutive ranges of 20%, and each quintile was analyzed separately according to the procedure described in the manuscript for the complete dataset. The results are presented in the Figure below.

As shown in Figure S14, panel A, the lower two quintiles of SupT1/CCR5 cells largely overlap with the range of CCR5 expression on primary T cells. We thus focused on the analysis of those two quintiles. Panels B and C in Figure S14 show how the phenotype maps calculated based on the lowest (B) or second lowest quintile (C) differ from the phenotype map based on the full range of CCR5 expression as presented in the manuscript. V3 variants are labelled and colored according to the phenotype reported in the manuscript based on the entire data set. The arrows connect the position of a variant on the initial map to the shifted position inferred from models based on a limited range of CCR5 expression. While some local rearrangements of single variants on the maps were observed, the same overall distribution of phenotypes and the same separation were maintained.

The slightly higher differences of clone positions on the map based on the lowest quintile (panel B) compared to the second lowest quintile (panel C) might be due to the limited number of cells in this cell subpopulation (only 7% of total cells on average, as compared to 37% for the second quintile, panel D). We therefore inspected how the shift of a position of an individual variant on the map varies depending on the number of cells from which this position is calculated. Panel E shows the relationship between the change of the correlation of a variant to the reference R5 and X4 models against the number of cells from which this position was inferred. There is a significant negative correlation indicating that the number of cells in a given quintile rather than the level of CCR5 expression might contribute to the change of the phenotype maps.

These results indicate the robustness of our approach towards various levels and various ranges of CCR5 expression and suggest the applicability of our results to different cell types.

| **A** | 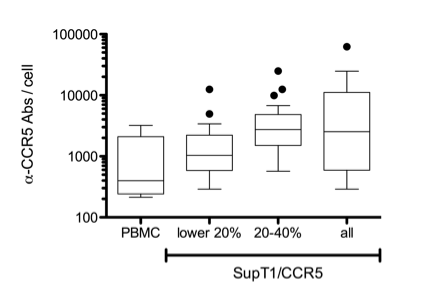 |
| --- | --- |
| 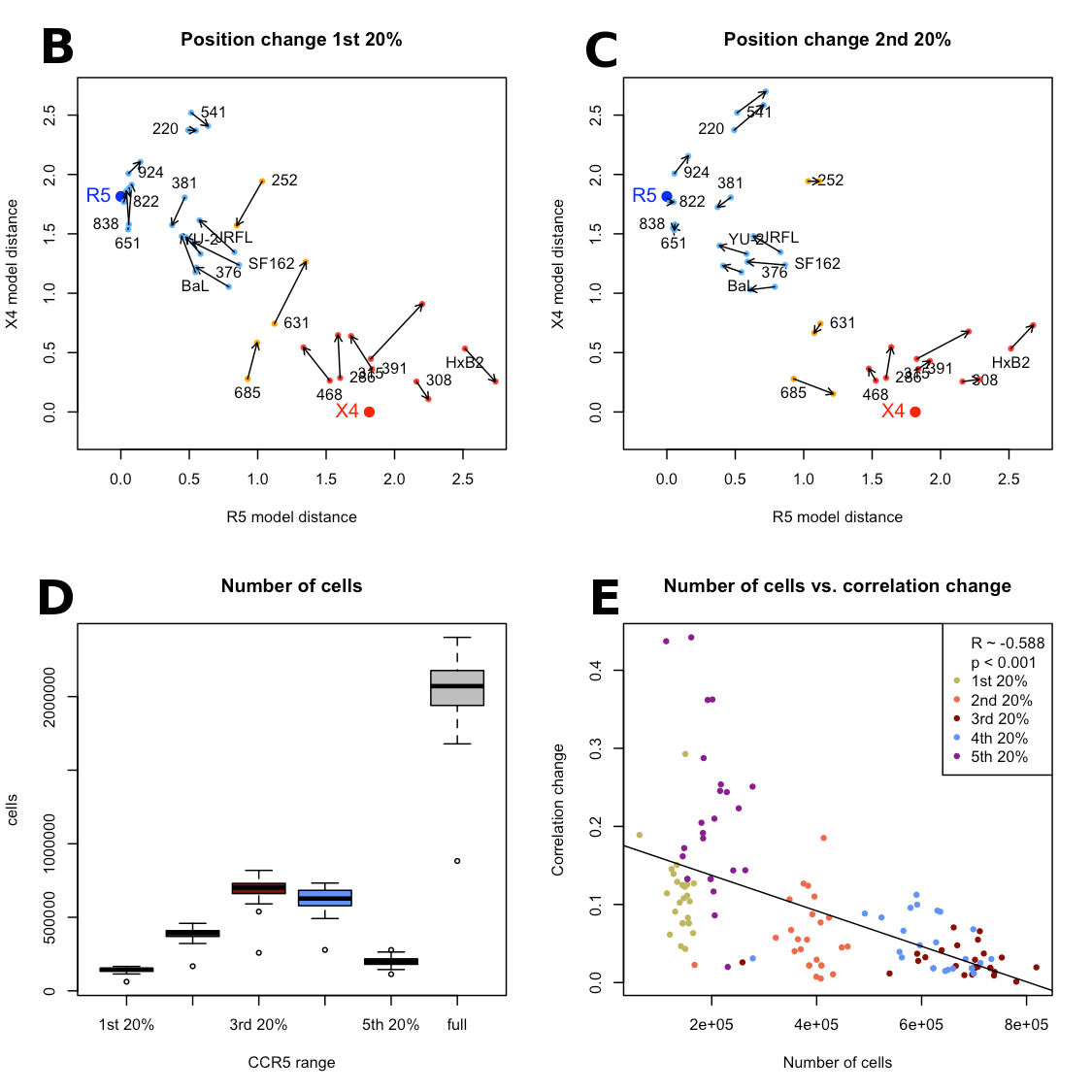 | |
| Figure S14. Model reproducibility on different CCR5 expression levels  **A. Range of lower two quintiles of CCR5 surface expression levels within the data set.**  Boxplots depict the range of CCR5 surface expression with Tukey whiskers on PBM cells and SupT1/CCR5 cell subpopulations. Data points above the 1.5 interquartile range are depicted as black dots. PBMCs were prepared from buffy coats from two (one experiment) to three (three experiments) donors and stimulated for three to five days using IL-2 (1µg/ml) and PHA (2µg/ml). Raw PE-staining values were determined by quantitative flow cytometry and numbers of antibodies bound per cell were estimated using the QuantiBrite PE kit (BD Biosciences) according to manufacturer’s protocol, assuming a PE:antibody ratio of 1:1. Median cell surface expression levels are depicted for PBMCs, whereas minimum and maximum values of single cell analyses of all experiments used for modeling in this study were taken into account for SupT1/CCR5 cells.  **B-C. Phenotype maps based on the lower two quintiles of the CCR5 expression levels.**  Phenotype vectors and phenotype maps were calculated based on the lower two quintiles (0-20% (A), 20-40% (B)). Virus variants are labeled, the color indicates their phenotype as reported in the manuscript: red for X4 variants, blue for R5 variants, orange for dual-tropic variants. The variants positions inferred from limited ranges of CCR5 expression are plotted against their positions inferred from the entire expression range as found in the manuscript. Arrows connect the original position of a variant on the map to its position inferred from the limited CCR5 expression range.  **D. Number of cells in each quantile of CCR5 expression range.**  **E. Relationship of variant position change on the map to the number of cells that the position is inferred from.**  The change between a limited and full range of CCR5 expression of a phenotype vectors correlation with the reference phenotype vectors is plotted against the number of cells that a given phenotype vector was calculated from. Each dot represents a variant in a given range of CCR5 expression as indicated by the dot coloring. Higher numbers of cells observed in the middle quintile of the expression range result in lower changes of variant position on the map. | |

REFERENCES

Bozek, K., A. Thielen, et al. (2009). "V3 loop sequence space analysis suggests different evolutionary patterns of CCR5- and CXCR4-tropic HIV." PLoS One **4**(10): e7387.

Fouchier, R. A., M. Groenink, et al. (1992). "Phenotype-associated sequence variation in the third variable domain of the human immunodeficiency virus type 1 gp120 molecule." J Virol **66**(5): 3183-3187.

Jensen, M. A. and A. B. van 't Wout (2003). "Predicting HIV-1 coreceptor usage with sequence analysis." AIDS Rev **5**(2): 104-112.

Shioda, T., J. A. Levy, et al. (1992). "Small amino acid changes in the V3 hypervariable region of gp120 can affect the T-cell-line and macrophage tropism of human immunodeficiency virus type 1." Proc Natl Acad Sci U S A **89**(20): 9434-9438.

Sing, T., A. J. Low, et al. (2007). "Predicting HIV coreceptor usage on the basis of genetic and clinical covariates." Antivir Ther **12**(7): 1097-1106.

Tibshirani, R. (1996). "Regression shrinkage and selection via the lasso." J. R. Statist. Soc **B 58**: 267-288.

Tychonoff, A. (1943). "On the stability of inverse problems." Doklady Akademii Nauk SSSR **39**(5): 195-198.
